# Synthesis of acylglycerol derivatives by mechanochemistry

**DOI:** 10.3762/bjoc.15.78

**Published:** 2019-03-29

**Authors:** Karen J Ardila-Fierro, Andrij Pich, Marc Spehr, José G Hernández, Carsten Bolm

**Affiliations:** 1 Institute of Organic Chemistry, RWTH Aachen University, Landoltweg 1, D-52074 Aachen, Germany; 2 Functional and Interactive Polymers, Institute of Technical and Macromolecular Chemistry, RWTH Aachen University, Worringerweg 2, 52074 Aachen, Germany; 3 DWI-Leibniz-Institute for Interactive Materials, Forckenbeckstrasse 50, D-52074 Aachen, Germany; 4 Aachen Maastricht Institute for Biobased Materials (AMIBM), Maastricht University, Brightlands Chemelot Campus, Urmonderbaan22, 6167 RD Geleen, The Netherlands; 5 Department of Chemosensation, Institute for Biology II, RWTH Aachen University, D-52074 Aachen, Germany

**Keywords:** ball mill, coumarin, diacylglycerols, lipids, mechanochemistry

## Abstract

In recent times, many biologically relevant building blocks such as amino acids, peptides, saccharides, nucleotides and nucleosides, etc. have been prepared by mechanochemical synthesis. However, mechanosynthesis of lipids by ball milling techniques has remained essentially unexplored. In this work, a multistep synthetic route to access mono- and diacylglycerol derivatives by mechanochemistry has been realized, including the synthesis of diacylglycerol-coumarin conjugates.

## Introduction

**Figure 1:**
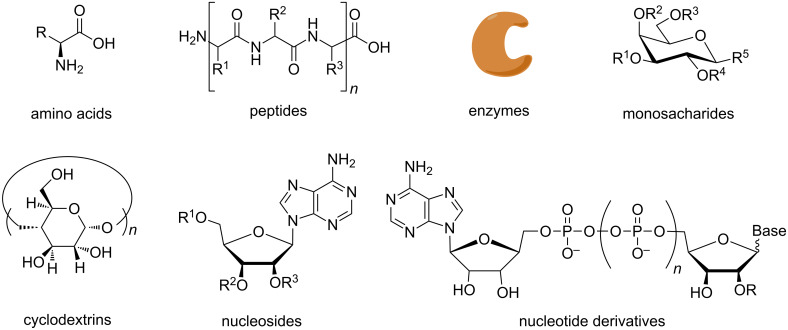
Biologically relevant molecules made, used or derivatized by mechanochemistry.

In addition to being guided by chemical signals, cells respond to mechanical cues by sensing and transducing external mechanical inputs into biochemical and electrical signals [1]. Consequently, every time a cell is subjected to mechanical loads, the biomolecules that constitute the cell do also experience the effects of the mechanical forces. For example, from the moment a nascent peptide begins growing in the ribosome, such peptide experiences mechanical signals that regulate the speed of protein synthesis [2]. Not surprisingly, the natural ability of peptides to endure mechanical stress in nature has allured scientists to evaluate the mechanical stability of proteins by using single-molecule nanomechanical techniques (e.g., magnetic and optical tweezers or atomic force microscopy) [3–4]. Additionally, the resilience of the peptide bond to mechanical loads has led to mechanoenzymatic transformations [5–7], and to synthesize amino acid derivatives [8–10] and peptides [11–13] by ball milling and extrusion techniques. Similarly, mechanochemical derivatizations of sugars and sugar derivatives such as cyclodextrins (CDs) have proven compatible with the use of ball mills. These reports showed advantages such as higher selectivity by ball milling compared to classic solution methods and the possibility to effectively react CDs and reactants of different solubility profiles [14–17]. The compatibility of synthesizing biologically relevant building blocks with mechanochemistry has further been shown by the recent mechanochemical protocols to transform nucleoside and nucleotide substrates (Figure 1) [18–19].

**Figure 2:**
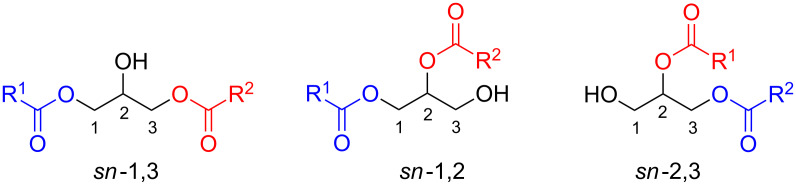
Isomeric diacyl-*sn*-glycerols (DAGs).

On the other hand, reports on mechanochemical protocols for the synthesis or derivatization of lipids are scarce [20–21]. Among the variety of amphipathic or hydrophobic small molecules that exhibit a lipid structure, diacylglycerols (DAGs) are important due to their signaling functions in cells (DAG signaling) [22–24]. Structurally, DAGs are glycerolipids containing two fatty acids esterified to the alcohol glycerol (Figure 2).

**Scheme 1:**
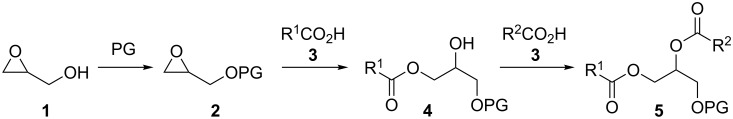
Synthetic route to access protected DAGs; PG = protecting group.

Biological routes that lead to the formation of DAGs include enzymatic degradation of glycerophospholipids and lipolysis of triacylglycerols (TAGs) [22,25]. However, due to the structural diversity of fatty acids present in acylglycerols and to the small structural differences among these fatty acids (e.g., chain length, degree of unsaturation, double bond position or stereochemistry), the access to pure DAGs and TAGs from natural sources by extraction is cumbersome. Alternatively, protected DAGs **5** can be chemically synthesized starting from glycerol [26] or glycidol [27], but either synthetic alternative involves multiple preparative steps in organic solvents (e.g., CH_2_Cl_2_, THF, Et_2_O). These considerations led us to explore a mechanochemical multistep route for the synthesis of protected DAGs **5** starting from glycidol (**1**) through the installation of a hydroxy protecting group, followed by epoxide ring-opening and esterification reactions with fatty acids **3** (Scheme 1).

If successful, developing a multistep approach to prepare DAGs would contribute to the expansion of synthetic mechanochemical methodologies in ball mills [28–31], which are often limited to single-step transformations. Additionally, synthesizing lipid structures mechanochemically would complement the preparation of biologically relevant building blocks (amino acids, peptides, saccharides, nucleosides, etc.) by mechanochemistry, thereby highlighting the importance of mechanical forces in the chemistry of life.

## Results and Discussion

**Scheme 2:**
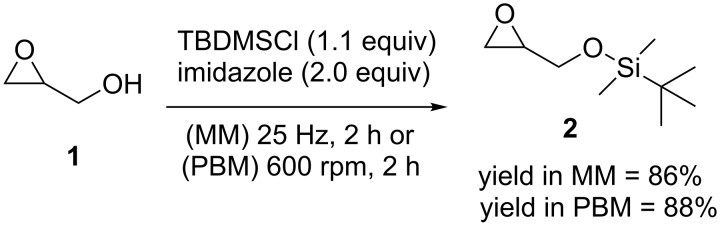
Protection of glycidol (**1**) with TBDMSCl in the ball mill. MM = mixer mill, PBM = planetary ball mill.

To commence, we focused on the synthesis of *tert*-butyldimethylsilyl glycidyl ether (**2**) by reacting glycidol (**1**; 0.67 mmol) and *tert*-butyldimethylsilyl chloride (TBDMSCl) in the presence of imidazole in a mixer mill (MM, Scheme 2) [32].

After 2 h of milling at 25 Hz full consumption of the starting materials was observed by thin-layer chromatography (TLC), with exclusive formation of the corresponding silyl ether **2**. However, the high volatility of **2** during posterior vacuum drying processes prevented the isolation of the TBDMS-protected glycidol **2** in higher yields after separation by column chromatography. A 5-fold scaled up version of the reaction using 3.37 mmol of **1** was carried out in a planetary ball mill (PBM) using 45 mL milling containers at 600 rpm, under otherwise identical conditions to afford product **2** in a similar yield.

**Scheme 3:**
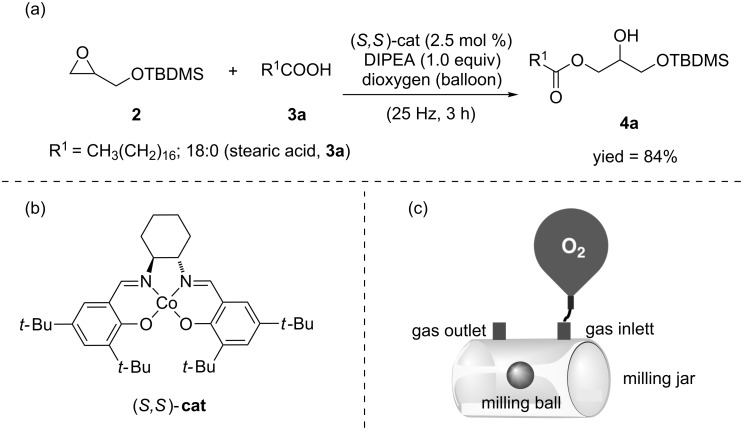
Cobalt-catalyzed epoxide ring-opening in the ball mill.

With TBDMS-protected glycidol **2** in our hands, a selective epoxide ring-opening reaction with fatty acids **3** leading to the formation of the corresponding *sn*-1,3-protected monoacylglycerols (MAGs **4**) was attempted (Scheme 3). Initially, commonly used solution-based protocols were tested in the ball mill [33]. For example, **2** was reacted with stearic acid (**3a**) in the presence of amines such as pyridine or tributylamine. However, the analysis of the reaction mixture only showed unreacted starting materials. In previous work, an acceleration of the oxirane ring-opening reaction with carboxylic acids [34] or alcohols [35] by using Lewis acid catalysts such as iron(III) chloride or bismuth(III) triflate was reported. However, the implementation of these protocols in the ball mill only led to trace amounts (less than 5% yield) of the protected monoacylglycerol **4a**. Finally, one of the well-established Jacobsen catalysts for the epoxide ring-opening reaction of **2** with stearic acid (**3a**) was evaluated (Scheme 3a).

Specifically, we focused on the use of Jacobsen cobalt(II)-salen complex (*S*,*S*)-**cat** (Scheme 3b), since similar salen complexes had originally been reported to facilitate epoxide ring-opening reactions with carboxylic acids as nucleophiles [36]. Moreover, salen complexes endure mechanochemical conditions, as proven during their preparation in ball mills [37]. In addition, various related Jacobsen salen complexes have shown catalytic activity under solvent-free conditions [38]. Collectively, these precedents made this synthetic route a promising one to mechanochemically access MAGs by ball milling.

Experimentally, we attempted the cobalt-catalyzed epoxide ring-opening reaction by milling **2** with stearic acid (**3a**) in the presence of (*S,S*)-**cat** (2.5 mol %) and *N*,*N*-diisopropylethylamine (DIPEA; 1.0 equiv, Scheme 3a). Mechanistically, it is known that Co(II) complex (*S,S*)-**cat** is catalytically inactive and its oxidation is required to facilitate the reaction [39]. Aware of this, we began by relying on the atmospheric dioxygen inside the milling container to oxidize complex (*S,S*)-**cat**. This approach has been applied before in the mechanochemical synthesis of Cu–carbene complexes from *N*,*N*-diarylimidazolium salts, dioxygen and metallic copper [40], which involved a mechanochemical reaction with gaseous reagents [41]. Pleasingly, this time the reaction afforded product **4a**, although the yield remained low (59%), even after three hours of milling. In order to improve the yield, we carried out the activation of the Co(II) complex (*S,S*)-**cat** by milling under a balloon pressure of dioxygen. Now, the yield of **4a** was boosted up to 84% yield (for a representation of the set up used and experimental details, see Scheme 3c and Supporting Information File 1). Analysis of the reaction mixture by ^1^H NMR revealed that the epoxide ring-opening had occurred preferentially to give *sn*-1,3-protected monoacylglycerol **4a** over its regioisomer counterpart *sn*-2,3-protected monoacylglycerol **4a’** (**4a**/**4a’** 3.6:1; for details, see Supporting Information File 1). However, purification of **4a** by column chromatography on SiO_2_ favored acyl migration in **4a**, thereby dropping the yield of **4a** by increasing the amount of the isomeric *sn*-2,3-protected monoacylglycerol **4a’** (Figure 2) [42]. Typically, cobalt complex (*S,S*)-**cat** has been used for kinetic resolution of racemic epoxides, for which the maximum theoretical yield of the reaction is 50%. Here, however, the yield of the *sn*-1,3-protected monoacylglycerol was high, and consequently we expected the enantiomeric excess of the product to be low. This, assumption was confirmed by analysis of the sample by high-performance liquid chromatography-chiral stationary phase (CSP-HPLC, for more details, see Supporting Information File 1). Access to enantiopure MAGs could be achieved under similar reaction conditions by starting from optically active commercially available silyl-protected glycidol derivatives [27,43].

**Scheme 4:**
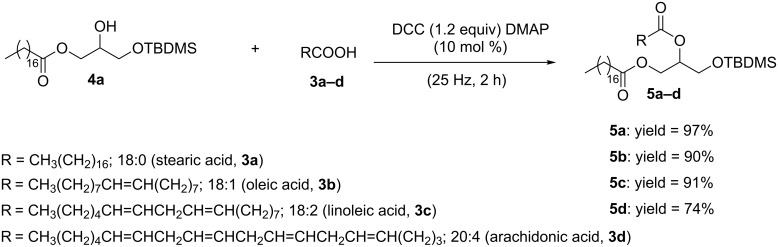
Mechanosynthesis of DAGs **5**.

Next, we targeted the mechanosynthesis of the DAGs by reacting MAG **4a** and fatty acids **3** in the ball mill. Such esterifications required the activation of **3** with *N*,*N*’-dicyclohexylcarbodiimide (DCC), thereby complementing other recently developed solvent-free carboxylic acid activations towards amidation or esterification reactions by ball milling [44]. In practice, we milled a mixture of MAG **4a**, stearic acid (**3a**), DCC and 4-dimethylaminopyridine (DMAP) at 25 Hz for 2 h in a mixer mill. Separation of the product by column chromatography gave DAG **5a** in 97% yield (Scheme 4).

**Scheme 5:**
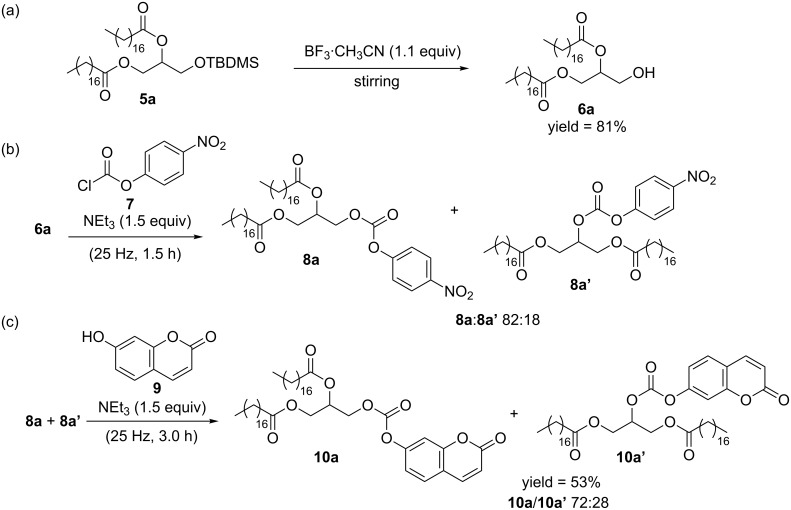
Conjugation of DAG **5a** with 7-hydroxycoumarin (**9**).

Alternatively, **5a** could be prepared following a one-pot two-step approach in the ball mill by beginning with the cobalt-catalyzed epoxide-ring opening of **2** with **3a**, followed immediately by the esterification of the corresponding MAG **4a** with stearic acid (**3a**), DCC and DMAP. Although successful, this strategy led to DAG **5a** in lower yield (50%). Then, in order to expand the library of DAGs, other fatty acids containing various degrees of unsaturation and chain length were tested. For instance, esterification of MAG **4a** with oleic acid (**3b**), linoleic acid (**3c**), and arachidonic acid (**3d**) underwent smoothly in the ball mill affording DAGs **5b**–**d** in yields up to 91% (Scheme 4). Particularly interesting was the formation of DAG (18:0/20:4) **5d**, an important lipidic backbone present in the biologically relevant phosphatidylinositol 4,5-bisphosphate (PIP2) [22]. In fact, diacylglycerols have proven to play vital roles in regulation of lipid bilayer and in the catalytic action of various membrane-related enzymes, such as protein kinase C (PKC) isoforms [45]. Therefore, the development of strategies for visualization of acylglycerols in cellular environments by their fusion with fluorescent molecular labels is in high demand [46]. As a result, once the mechanosynthesis of DAGs **5** was established, we turned our efforts towards the conjugation of DAG **5a** with 7-hydroxycoumarin (**9**) (Scheme 5).

**Figure 3:**
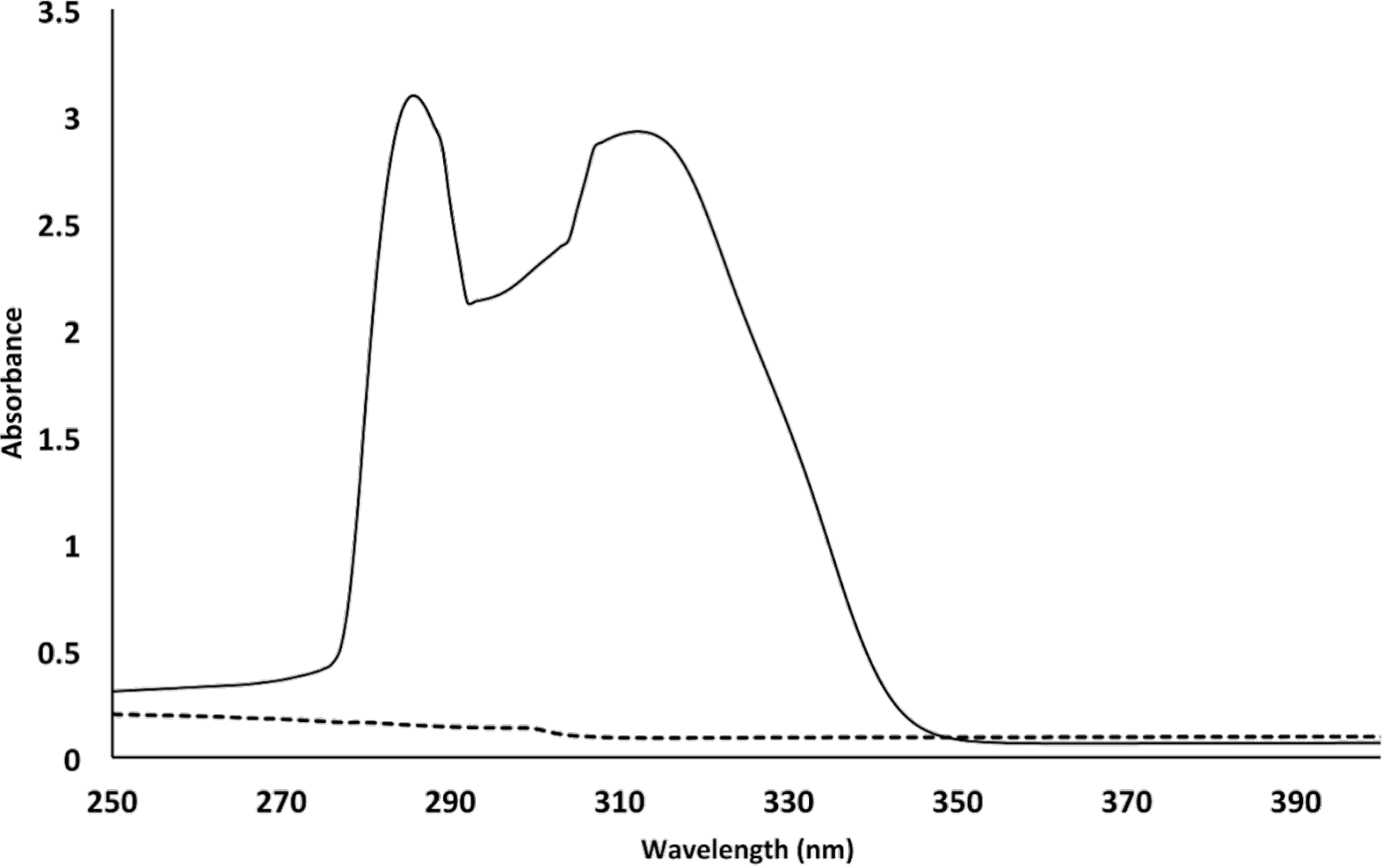
UV−vis spectra of DAG **6a** (dotted line) and conjugated DAGs **10a** and **10a’** as a mixture (**10a**/**10a’** 72:28) in toluene at a concentration of 0.5 μM.

Initially, removal of the TBDMS protecting group of **5a** was attempted by milling. However, reacting DAG **5a** with a mixture of BF_3_·CH_3_CN and silica gel followed by an aqueous work-up gave DAG **6a** in only 31% yield, together with concomitant acyl migration of the corresponding *sn*-1,2-diacylglycerol **6a** into *sn*-1,3-diacylglycerol **6a’**. Therefore, the desilylation reaction was carried out by stirring **5a** and BF_3_·CH_3_CN (Scheme 5a). Next, DAG **6a** was reacted with 4-nitrophenyl chloroformate (**7**) and triethylamine in the ball mill to form the activated DAG anhydride derivative **8a** (Scheme 5b). Analysis by ^1^H NMR spectroscopy of the reaction mixture revealed the presence of DAG **8a** along with its isomeric DAG **8a’**. Formation of the latter compound could have been facilitated through acyl migration of **6a** under the basic milling conditions. Subsequently, the reaction mixture containing **8a** and **8a’** was milled with 7-hydroxycoumarin (**9**) and triethylamine to achieve the conjugation of the DAGs **8** in 53% yield after two steps (Scheme 5c). A mixture of **10a** and **10a’** (**10a**/**10a’** 72:28) was separated from the unreacted starting materials, and analyzed by UV–vis spectroscopy (Figure 3). Comparison of the UV–vis spectra of **6a** and **10a**/**10a’** showed the successful conjugation of the DAG with the coumarin moiety [47].

## Conclusion

The implementation of ball milling techniques has provided the opportunity to extend the applicability of mechanochemistry to the synthesis of architecturally complex targets, such as mono- and diacylglycerols. Altogether, the mechanosynthesis of lipids and lipid derivatives complements the current systematic work towards the synthesis of other biologically relevant molecules under environments of high mechanical stress. Specifically, the synthesis of mono- and diacylglycerols required first, the application of solventless functional group protection chemistry in ball mills, second, the implementation of metal-catalyzed epoxide-ring opening, and third, the development of solvent-free ester formation between monoacylglycerols and fatty acids to afford DAGs. Moreover, the synthesis of conjugated DAGs **10** represents a step forward towards the establishment of mechanochemical conjugation reactions for linking fluorescent materials to lipids at the proof-of-concept level.

## Supporting Information

File 1 Experimental procedures, set-ups and characterization data.
