# Supplementary material for: Synthesis of acylglycerol derivatives by mechanochemistry
Source: Beilstein J Org Chem. 2019 Mar 29;15:811–7. doi: 10.3762/bjoc.15.78 (PMC6444433; doi:10.3762/bjoc.15.78)
Supplement: File 1 — Experimental procedures, set-ups and characterization data. [file Beilstein_J_Org_Chem-15-811-s001.pdf]

BEILSTEIN JOURNAL OF ORGANIC CHEMISTRY

Supporting Information

for

Synthesis of acylglycerol derivatives by mechanochemistry

Karen J. Ardila-Fierro, Andrij Pich, Marc Spehr, José G. Hernández and Carsten Bolm

Beilstein J. Org. Chem. **2019,** *15,* 811–817. doi:10.3762/bjoc.15.78

Experimental procedures, set-ups and characterization data

License and Terms: This is a supporting information file under the terms of the Creative Commons Attribution License (http://creativecommons.org/licenses/by/4.0). Please note that the reuse, redistribution and reproduction in particular requires that the authors and source are credited.
The license is subject to the *Beilstein Journal of Organic Chemistry* terms and conditions: (https://www.beilstein-journals.org/bjoc)

CONTENTS

1. General information S2

2. General procedures in the ball mill S3

2.1 Mechanosynthesis of 2 S3

2.2 Mechanosynthesis of 4a S3

2.3 Mechanosynthesis of 5a–d S5

2.4 Deprotection of 5a S5

2.5 Mechanosynthesis of 8a and 8a’ S5

2.6 Mechanosynthesis of 10a and 10a’ S6

3. Table S1. Comparison between reported methodologies and the mechanochemical approach reported in this work S7

4. Characterization of the products S8

5. NMR spectra S14

6. Chiral stationary phase high-performance liquid chromatography (CSP-HPLC) analysis S23

7. References S24

1. General information

All chemicals are commercial available and they were used as received unless otherwise stated. Thin-layer chromatography (TLC) was performed using TLC plates (silica gel 60 on aluminum or glass with fluorescence indicator F254) from MERCK. Qualitative analysis of the TLC plates was carried out using UV light (λ = 254 nm and λ = 366 nm) and/or by immersion in an aqueous solution of potassium permanganate ($KMnO_4$) and heating of the stained plates with a heat-gun at 300 °C until dryness. Products were purified by column chromatography using silica gel 60 (40−63 μm) from ACROS Organics. Solvents for column chromatography were distilled prior to use.

All NMR spectra were recorded on a VNMRS 400 or on a VNMRS 600 spectrometer. Proton chemical shifts are reported in parts per million on the δ scale and are calibrated using the residual non-deuterated solvent signal as an internal reference. Spectral data is provided as follows: chemical shift in ppm (from downfield to upfield), multiplicity (s = singlet, d = doublet, m = multiplet), integration and coupling constant *J*.

High-resolution mass spectra were recorded on a THERMO FISHER Scientific Orbitrap XL spectrometer. UV–vis spectra were measured on a Shimadzu UV-

2600 spectrophotometer. Analytical HPLC measurements for the determination of the enantiomer ratios were performed on an Agilent 1100 using a CHIRALPAK® OD-H column (210 mm in length, 4.6 mm in internal diameter) and a mixture of *n*-heptane:IprOH 98:2 as eluent. The flow of the mobile phase was kept at 0.7 mL/min at 20 °C. Detection of the analytes was done at 210 nm.
Mechanochemical reactions were carried out in a RETSCH MM400 mixer mill or in a FRITSCH planetary micro mill model "Pulverisette 7 classic line".

2. General procedures in the ball mill

2.1 Mechanosynthesis of 2

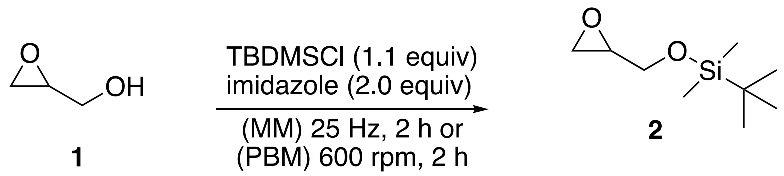

A mixture of **1** (50 mg, 0.67 mmol), *tert*-butylchlorodimethylsilane (0.73 mmol), and imidazole (1.34 mmol) was mixed in a 10 mL ZrO_2 milling jar with one ZrO_2 ball of 10 mm in diameter at 25 Hz for 2 h. After the milling was stopped, the reaction mixture was recovered from the milling jar and the product was purified by column chromatography (SiO_2, eluent 50:1 *n*-pentane/ethyl acetate). After purification, the product was obtained as a colorless oil in 47% yield. Compound **2** is prone to evaporation; therefore the yield of the reaction was also determined by ^{1}H NMR spectroscopy of the reaction mixture using as an internal standard 1,3,5 trimethoxybenzene, which showed **2** in 86% yield.

An scale up of the reaction using **1** (250 mg, 3.37 mmol), *tert*-butylchloridimethylsilane (560 mg, 3.71 mmol), and imidazole (460 mg, 6.74 mmol) was carried out in a planetary ball mill, using 45 mL ZrO_2 milling jars with five ZrO_2 balls of 10 mm in diameter at 600 rpm for 2 h. Analysis of the reaction mixture by ^{1}H NMR spectroscopy indicated the presence of **2** (558 mg) in 88% yield.

2.2 Mechanosynthesis of 4a

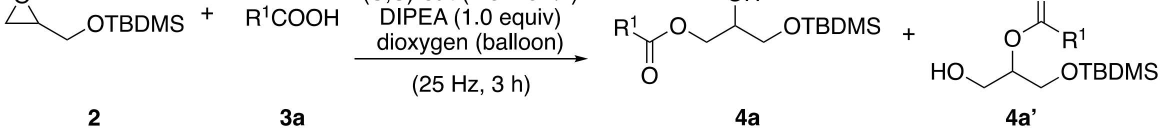

$R^1 = CH_3(CH_2)_{16}$; 18:0 (stearic acid, **3a**)

A mixture of (*S,S*)-**cat** (2.5 mol %, 0.02 mmol) and **3a** (226.5 mg, 0.796 mmol) was charged in a 20 mL stainless-steel milling jar with one 10 mm ball of the same

material. Initially, a gas balloon containing dioxygen was attached to the gas inlet on the milling jar and a continuous flow of dioxygen was kept for 1 min. Then the gas outlet was closed with a plastic screw to prevent leaking of the reaction mixture and the content was mixed for 15 min at 25 Hz under oxygen atmosphere (Figure S1). After 15 min of milling the dioxygen flow was disrupted by removing the balloon. Then, DIPEA (0.796 mmol) was added and the gas inlet was closed with a plastic screw. Next, the mixture was milled for another 10 min at 25 Hz. Finally, **2** (150 mg, 0.796 mmol) was added into the jar and the content was milled at 25 Hz for 155 min. Once the milling was stopped, the reaction mixture was analyzed by ^{1}H NMR spectroscopy using 1,3,5-trimethoxybenzene as an internal standard. The NMR analysis showed full conversion of the starting material and the presence of a mixture of products **4a** and **4a’** in a 4:1 ratio. Purification of the products by column chromatography (SiO_2, eluent 40:1 *n*-pentane:ethyl acetate) afforded **4a** as a white-yellowish solid in 42% yield.

At no point during this research project we noticed any exothermicity of mechanochemical reactions upon milling under oxygen atmosphere. However, it is important to highlight that some substances react violently with oxygen, which can lead to either combustion after ignition or even self-ignition. Therefore, extra precaution is recommended when reacting organic molecules in oxygen systems using stainless-steel milling media.

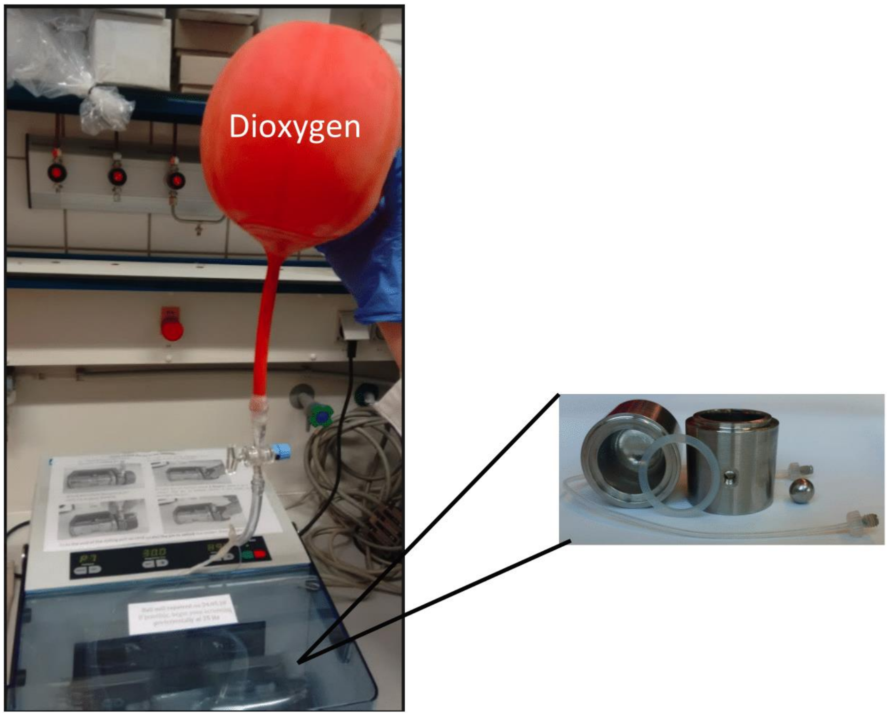

Figure S1: Setup for cobalt-catalyzed epoxide-ring opening in the mixer mill highlighting a modified 20 mL stainless-steel milling jar with a gas inlet and a gas outlet.

2.3 Mechanosynthesis of 5a–d

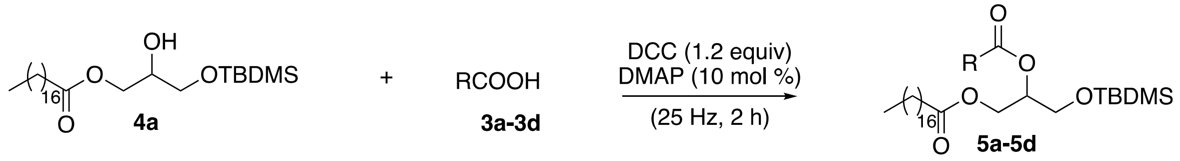

5a; yield = 97%
5b; yield = 90%
5c; yield = 91%
5d; yield = 74%

R = $CH_3(CH_2)_{16}$; 18:0 (stearic acid, **3a**)

R = $CH_3(CH_2)_7CH{=}CH(CH_2)_7$; 18:1 (oleic acid, **3b**)

R = $CH_3(CH_2)_4CH{=}CHCH_2CH{=}CH(CH_2)_7$; 18:2 (linoleic acid, **3c**)

R = $CH_3(CH_2)_4CH{=}CHCH_2CH{=}CHCH_2CH{=}CHCH_2CH{=}CH(CH_2)_3$; 20:4 (arachidonic acid, **3d**)

A mixture of **4a** (204.9 mg, 0.433 mmol), fatty acid **3** (**3a–d**, 0.520 mmol), DCC (0.520 mmol) and 4-DMAP (10 mol %, 0.0433 mmol) was milled using a 10 mL ZrO_2 milling jar and one 10 mm ball of the same material at 25 Hz for 2 h. The products **5a–d** were purified by column chromatography (SiO_2, eluent 80:1 *n*-pentane/ethyl acetate).

2.4 Deprotection of 5a

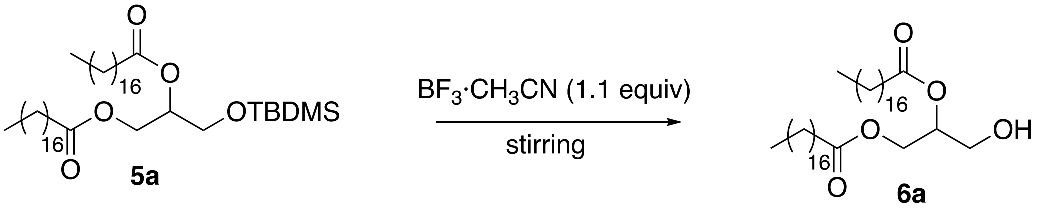

Compound **5a** (251.4 mg, 0.34 mmol) was dissolved in DCM or CH_3CN (3 mL) at 0 °C, followed by addition of $BF_3{\cdot}CH_3CN$ (0.37 mmol), the reaction mixture was stirred for 7 min until complete conversion of **5a** was confirmed by thin-layer chromatography. The product **6a** was isolated after extraction in 81% yield [1].

2.5 Mechanosynthesis of 8a

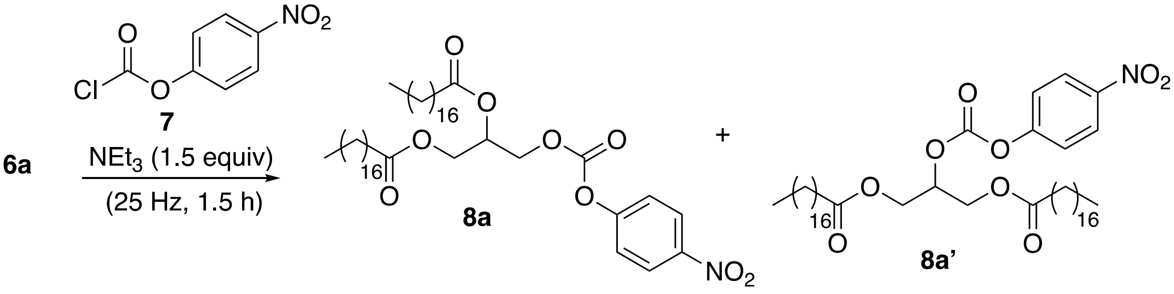

8a:**8a'** 82:18

A mixture of **6a** (159.2 mg, 0.254 mmol), **7** (0.254 mmol) and NEt_3 (0.382 mmol)

was milled in a 10 mL ZrO_2 milling jar with one 10 mm ball of the same material at 25 Hz for 90 min. Analysis by ^1H NMR spectroscopy showed full consumption of **6a** after 90 min of milling. Therefore, the reaction mixture was used in the next step without further purification.

2.6 Mechanosynthesis of 10a and 10a'

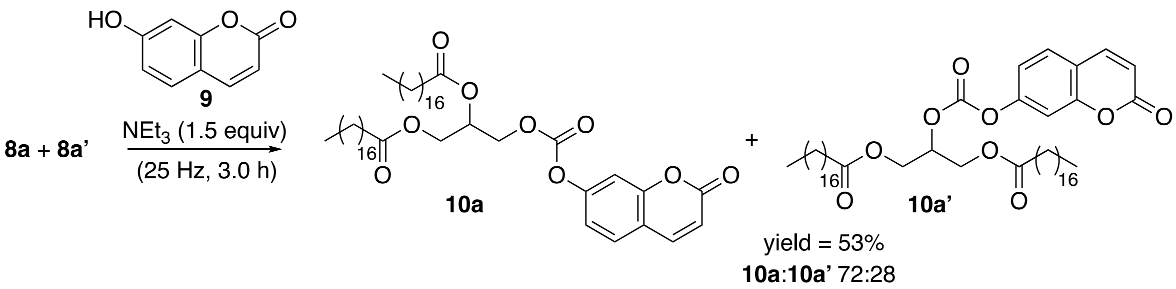

To the reaction mixture containing **8a** and **8a'** (section 2.5), **9** (41.35 mg, 0.254 mmol) and NEt_3 (0.382 mmol) were added. This mixture was milled at 25 Hz for 3 h. Once the milling was stopped, the reaction mixture was analyzed by ^1H NMR spectroscopy using 1,3,5-trimethoxybenzene as an internal standard. The NMR analysis showed the presence of DAGs **10a** and **10a'** in a 2.5:1 ratio. Products **10a** and **10a'** were inseparable by column chromatography (SiO_2, eluent 10:1 *n*-pentane/ethyl acetate). However, a mixture containing exclusively DAG **10a** and **10a'** was obtained in 53% yield.

3. Table S1. Comparison between reported methodologies and the mechanochemical approach reported in this work

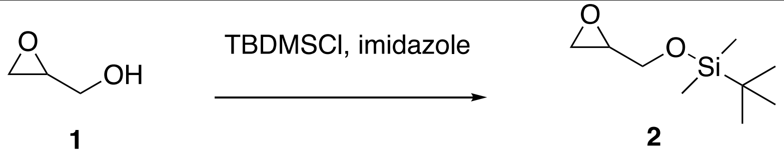

Method	Solvent and additives	Reaction time	Yield	References
Solution	CH_2Cl_2 DMAP	Overnight 0°C to rt	96%	Org. Lett. **2013**, 15, 1424–1427
Solution	THF	Overnight rt	100%	*Org. Biomol. Chem*. **2011**, *9*, 8030–8037
Ball milling	---	2 h	86%	This work

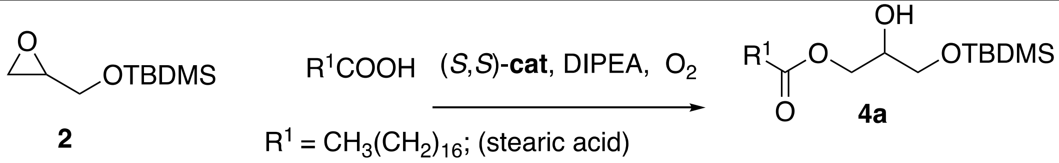

Method	Solvent	Reaction time	Yield	References and notes
Neat/stirring	---	16 h	NA	*Org, Biomol. Chem.* **2013**, *11*, 6919−6928<br>-(*R*)-TBDMS-glycidyl ether **2** was used.<br>-Yield for **4a** was not reported, but **4a** was used in a sequential reaction leading to the formation of a subsequent product in 91%.
Ball milling	---	3 h	84%	This work

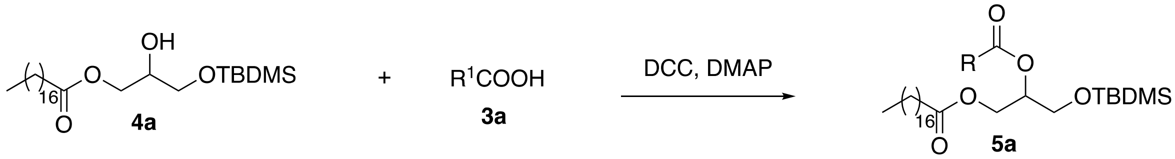

Method	Solvent	Reaction time	Yield	References and notes
Solution	Heptane	16 h	91%	*Org, Biomol. Chem.* **2013**, *11*, 6919−6928<br>-R^1 = $CH_3(CH_2)_{14}$; (palmitic acid)<br>-(*R*)-**4a** was used.
Ball milling	---	2 h	97%	This work<br>-R^1 = $CH_3(CH_2)_{16}$; (stearic acid)

4. Characterization of the products

tert-Butyldimethylsilyl-glycidyl ether (2)

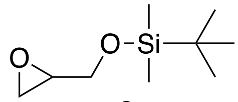

2

Molecular formula: $C_9H_{20}O_2Si$
Molecular Mass: 188.3420 g/mol
Physical appearance: colorless oil
TLC: *Rf* (silica gel) = 0.58 (*n*-pentane/EtOAc 10:1); KMnO4 active.

^{1}H NMR (400 MHz, $CDCl_3$), δ (ppm): 3.85 (dd, *J* = 11.9 Hz, *J* = 3.2 Hz, 1H, CH_2), 3.66 (dd, *J* = 11.9 Hz, *J* = 4.8 Hz, 1H, CH_2), 3.10-3.06 (m, 1H, CH), 2.76 (dd, *J* = 5.0 Hz, *J* = 4.2, 1H, CH_2), 2.63 (dd, *J* = 5.2 Hz, *J* = 2.7 Hz, 1H, CH_2), 0.90 (s, 9H, ^{t}Bu), 0.08 (s, 3H, CH_3), 0.07 (s, 3H, CH_3).

^{13}C NMR (100 MHz, $CDCl_3$), δ (ppm): 63.8, 52.5, 44.6, 26.0, 18.5, −5.1, −5.2. The spectral data for this compound match that reported in the literature [2].

1-Stearoyl-3-(*tert*-butyldimethylsilyl)-*sn*-glycerol (4a)

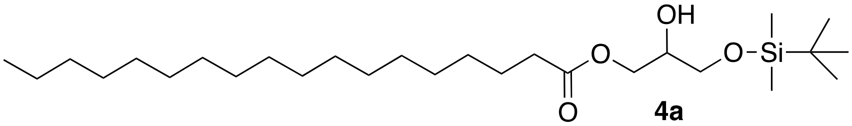

4a

Molecular formula: $C_{27}H_{56}O_4Si$
Molecular Mass: 472.8260 g/mol
Physical appearance: white-yellowish solid
TLC: *Rf* (silica gel) = 0.32 (*n*-pentane/EtOAc 10:1); KMnO4 active.

^{1}H NMR (400 MHz, $CDCl_3$), δ (ppm): 4.17-4.08 (m, 2H, CH_2), 3.90-3.84 (m, 1H, CH), 3.67 (dd, *J* = 10.1 Hz, *J* = 4.6 Hz, 1H, CH_2), 3.60 (dd, *J* = 10.1 Hz, *J* = 5.6 Hz, 1H, CH_2), 2.76 (dd, *J* = 5.0 Hz, *J* = 4.2, 1H, CH_2), 2.50 (br s, 1H, OH), 2.33 (t, *J* = 7.6 Hz, 2H, CH_2), 1.66-1.58 (m, 2H, CH_2), 1.28-1.25 (m, 28H, 14 x CH_2), 0.90-0.86 (m, 12H, 4 x CH_3), 0.07 (s, 6H, 2 x CH_3).

^{13}C NMR: (100 MHz, $CDCl_3$), δ (ppm): 174.1, 70.1, 65.1, 63.8, 34.3, 32.0, 29.8, 29.8, 29.8, 29.7, 29.6, 29.5, 29.4, 29.3, 25.9, 25.1, 22.8, 18.4, 14.2, −5.3, −5.3.

HRMS (ESI$^+$) m/z: calcd. for $[M+Na]^+$ = $[C_{27}H_{56}O_4NaSi]^+$: 495.38400; found: 495.38401. The spectral data for this compound match that reported in the literature [3].

1,2-Distearoyl-3-(*tert*-butyldimethylsilyl)-*sn*-glycerol (5a)

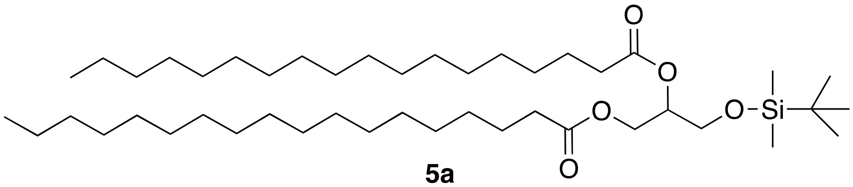

5a

Molecular formula: $C_{45}H_{90}O_5Si$
Molecular Mass: 738.6558 g/mol
Physical appearance: white solid
TLC: *Rf* (silica gel) = 0.72 (*n*-pentane/EtOAc 10:1); KMnO4 active.
^{1}H NMR (400 MHz, $CDCl_3$), δ (ppm): 5.09-5.06 (m, 1H, CH), 4.34 (dd, *J* = 11.9 Hz, *J* = 3.7 Hz, 1H, CH_2), 4.16 (dd, *J* = 11.9 Hz, *J* = 6.2 Hz, 1H, CH_2), 3.72-3.70 (m, 2H, CH_2), 2.30 (t, *J* = 7.4 Hz 4H, 2 x CH_2), 1.63-1.57 (m, 4H, 2 x CH_2), 1.30-1.25 (m, 56H, 28 x CH_2), 0.88-0.86 (m, 15H, 5 x CH_3), 0.06-0.04 (m, 6H, 2 x CH_3).
^{13}C NMR: (100 MHz, $CDCl_3$), δ (ppm): 173.4, 173.0, 71.6, 62.4, 61.4, 34.3, 34.1, 31.9, 29.6, 29.6, 29.6, 29.4, 29.3, 29.2, 29.1, 29.0, 25.7, 24.9, 24.8, 22.6, 18.1, 14.0, −5.5, −5.5.
HRMS (ESI$^+$) m/z: calcd. for $[M+K]^+$ = $[C_{45}H_{90}O_5KSi]^+$: 777.61780; found: 777.61891. The spectral data for this compound match that reported in the literature [3].

1-Stearoyl-2-oleoyl-3-(*tert*-butyldimethylsilyl)-*sn*-glycerol (5b)

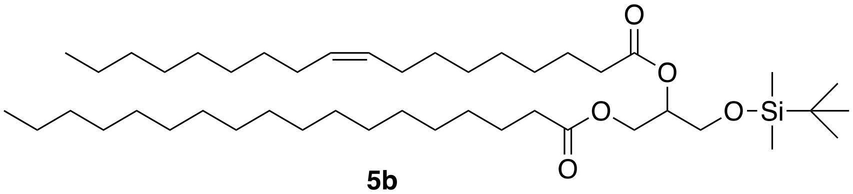

5b

Molecular formula: $C_{45}H_{88}O_5Si$
Molecular Mass: 736.6401 g/mol
Physical appearance: colorless oil
TLC: *Rf* (silica gel) = 0.73 (*n*-pentane/EtOAc 9:1); KMnO4 active.
^{1}H NMR (400 MHz, $CDCl_3$), δ (ppm): 5.36-5.32 (m, 2H, 2 x *H*C=C), 5.09-5.04 (m, 1H, CH), 4.34 (dd, *J* = 11.8 Hz, *J* = 3.6 Hz, 1H, CH_2), 4.14 (dd, *J* = 11.9 Hz, *J* = 6.3 Hz, 1H, CH_2), 3.72-3.70 (m, 2H, CH_2), 2.32-2.28 (m, 4H, 2 x CH_2), 2.04-1.98 (m, 4H, 2 x CH_2), 1.63-1.57 (m, 4H, 2 x CH_2), 1.34-1.25 (m, 48H, 24 x CH_2), 0.89-0.86 (m, 15H, 5 x CH_3), 0.05 (s, 6H, 2 x CH_3).
^{13}C NMR: (100 MHz, $CDCl_3$), δ (ppm): 173.4, 173.0, 129.9, 129.6, 71.6, 62.4, 61.4, 34.3, 34.1, 31.8, 29.6, 29.6, 29.6, 29.5, 29.4, 29.3, 29.2, 29.1, 29.1, 29.0, 27.1, 25.7, 24.9, 22.6, 18.1, 14.0, −5.5, −5.5.

HRMS (ESI$^+$) m/z: calcd. for $[M+Na]^+$ = $[C_{45}H_{88}O_5NaSi]^+$: 759.62939; found: 759.62932.

1-Stearoyl-2-linoleoyl-3-(*tert*-butyldimethylsilyl)-*sn*-glycerol (5c)

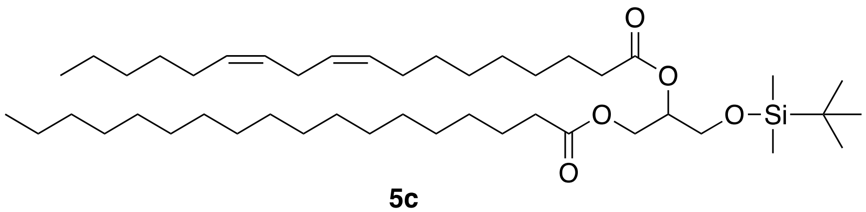

5c

Molecular formula: $C_{45}H_{86}O_5Si$
Molecular Mass: 734.6245 g/mol
Physical appearance: colorless oil
TLC: *Rf* (silica gel) = 0.75 (*n*-pentane/EtOAc 9:1); KMnO4 active.

^{1}H NMR (600 MHz, $CDCl_3$), δ (ppm): 5.39-5.30 (m, 4H, 4 x *H*C=C), 5.09-5.05 (m, 1H, CH), 4.34 (dd, *J* = 11.8 Hz, *J* = 3.6 Hz, 1H, CH_2), 4.16 (dd, *J* = 11.9 Hz, *J* = 6.3 Hz, 1H, CH_2), 3.72-3.71 (m, 2H, CH_2), 2.77 (t, *J* = 6.9 Hz, 2H, CH_2), 2.32-2.28 (m, 4H, 2 x CH_2), 2.06-2.03 (m, 4H, 2 x CH_2), 1.63-1.59 (m, 4H, 2 x CH_2), 1.34-1.25 (m, 42H, 21 x CH_2), 0.89-0.86 (m, 15H, 5 x CH_3), 0.05 (s, 6H, 2 x CH_3).

^{13}C NMR: (150 MHz, $CDCl_3$), δ (ppm): 173.4, 173.0, 130.2, 129.9, 128.0, 127.8, 71.6, 62.4, 61.4, 34.3, 34.1, 31.9, 31.5 29.7, 29.6, 29.6, 29.6, 29.4, 29.3, 29.3, 29.2, 29.2, 29.1, 29.0, 27.1, 25.7, 25.6, 24.9, 22.6, 22.5, 18.2, 14.1, 14.0, −5.4, −5.5.

HRMS (ESI$^+$) m/z: calcd. for $[M+Na]^+$ = $[C_{45}H_{86}O_5NaSi]^+$: 757.61389; found: 757.61367.

1-Stearoyl-2-arachidonoyl-3-(*tert*-butyldimethylsilyl)-*sn*-glycerol (5d)

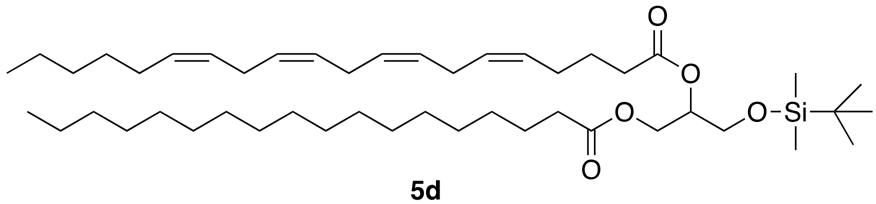

5d

Molecular formula: $C_{47}H_{86}O_5Si$
Molecular Mass: 758.6245 g/mol
Physical appearance: colorless oil
TLC: *Rf* (silica gel) = 0.67 (*n*-pentane/EtOAc 9:1); KMnO4 active.

^{1}H NMR (400 MHz, $CDCl_3$), δ (ppm): 5.39-5.30 (m, 8H, 8 x *H*C=C), 5.07-5.04 (m, 1H, CH), 4.32 (dd, *J* = 11.8 Hz, *J* = 3.7 Hz, 1H, CH_2), 4.14 (dd, *J* = 11.9 Hz, *J* = 6.2 Hz, 1H, CH_2), 3.70-3.68 (m, 2H, CH_2), 2.83-2.77 (m, 6H, 3 x CH_2), 2.32-2.26 (m,

4H, 2 x CH_2), 2.12-2.01 (m, 4H, 2 x CH_2), 1.72-1.61 (m, 4H, 2 x CH_2), 1.29-1.23 (m, 34H, 17 x CH_2), 0.88-0.84 (m, 15H, 5 x CH_3), 0.03 (s, 6H, 2 x CH_3).

^{13}C NMR: (100 MHz, $CDCl_3$), δ (ppm): 173.4, 172.7, 130.4, 128.8, 128.8, 128.5, 128.2, 128.0, 127.8, 127.4, 71.7, 62.3, 61.3, 34.3, 34.1, 31.8, 31.4, 29.6, 29.6, 29.6, 29.4, 29.3, 29.2, 29.2, 29.1, 29.0, 27.1, 26.4, 25.7, 25.6, 25.5, 24.8, 24.7, 22.6, 22.5, 18.1, 14.0, 14.0, −5.5, −5.5.

HRMS (ESI$^+$) m/z: calcd. for $[M+Na]^+$ = $[C_{47}H_{86}O_5NaSi]^+$: 781.61340; found: 781.61367. The spectral data for this compound match that reported in the literature [4].

1,2-Distearoyl-*sn*-glycerol (6a)

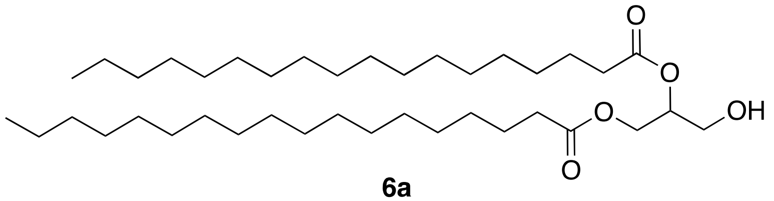

6a

Molecular formula: $C_{39}H_{76}O_5$

Molecular Mass: 624.5693 g/mol

Physical appearance: white solid

TLC: *Rf* (silica gel) = 0.6 (*n*-pentane/EtOAc 2:1); 0.3 (*n*-pentane/EtOAc 9:1); $KMnO_4$ active.

^{1}H NMR (400 MHz, $CDCl_3$), δ (ppm): 5.09-5.07 (m, 1H, CH), 4.32 (dd, *J* = 11.7 Hz, *J* = 4.6 Hz, 1H, CH_2), 4.24 (dd, *J* = 11.8 Hz, *J* = 5.6 Hz, 1H, CH_2), 3.74-3.72 (m, 2H, CH_2), 2.36-2.30 (m, 4H, 2 x CH_2), 2.03 (br s, 1H, OH), 1.65-1.58 (m, 4H, 2 x CH_2), 1.32-1.25 (m, 56H, 28 x CH_2), 0.89-0.86 (m, 6H, 2 x CH_3).

^{13}C NMR: (100 MHz, $CDCl_3$), δ (ppm): 173.7, 173.3, 72.0, 61.9, 61.5, 34.2, 34.0, 31.8, 29.6, 29.6, 29.5, 29.4, 29.3, 29.2, 29.0, 29.0, 24.9, 24.8, 22.6, 14.0.

HRMS (ESI$^+$) m/z: calcd. for $[M+Na]^+$ = $[C_{39}H_{76}O_5Na]^+$: 647.55743; found: 647.55850. The spectral data for this compound match that reported in the literature [1].

1,2-Distearoyl-3-(((4-nitrophenoxy)carbonyl)oxy)-*sn*-glycerol (8a)

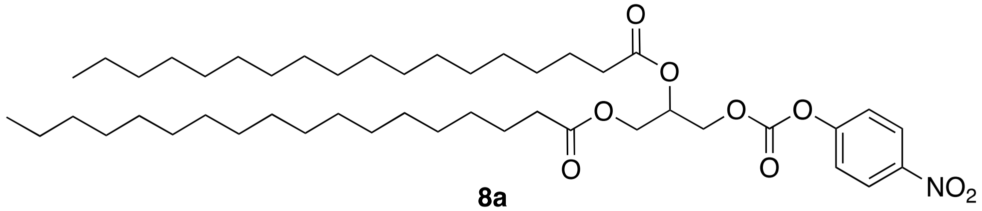

8a

Molecular formula: $C_{46}H_{79}NO_9$
Molecular Mass: 789.5755 g/mol
Physical appearance: white-yellowish solid
TLC: *Rf* (silica gel) = 0.78 (*n*-pentane/EtOAc 3:1); UV (254nm) and KMnO4 active.
^{1}H NMR (400 MHz, $CDCl_3$), δ (ppm): 8.29 (dt, *J* = 9.3 Hz, *J* = 2.1 Hz, 2H, 2 x CH), 7.39 (dt, *J* = 9.2 Hz, *J* = 2.2 Hz, 2H, 2 x CH), 5.40-5.35 (m, 1H, CH), 4.50 (dd, *J* = 11.7 Hz, *J* = 3.7 Hz, 1H, CH_2), 4.38-4.34 (m, 2H, CH_2), 4.22 (dd, *J* = 11.9 Hz, *J* = 5.6 Hz, 1H, CH_2), 2.38-2.30 (m, 4H, 2 x CH_2), 1.66-1.57 (m, 4H, 2 x CH_2), 1.32-1.24 (m, 56H, 28 x CH_2), 0.89-0.86 (m, 6H, 2 x CH_3).
^{13}C NMR: (100 MHz, $CDCl_3$), δ (ppm): 173.1, 172.8, 155.3, 152.2, 145.5, 125.3, 121.7, 68.2, 66.9, 61.5, 34.1, 33.9, 31.8, 29.6, 29.6, 29.5, 29.4, 29.3, 29.2, 29.0, 29.0, 24.8, 22.6, 14.0.
HRMS (ESI$^+$) m/z: calcd. for $[M+Na]^+$ = $[C_{46}H_{79}O_9NNa]^+$: 812.56848; found: 812.56470.

Mixture containing 1,2-distearoyl-3-((((2-oxo-2*H*-chromen-7-yl)oxy)carbonyl)oxy)-*sn*-glycerol (10a) and 1,3-distearoyl-2-((((2-oxo-2*H*-chromen-7-yl)oxy)carbonyl)oxy)-*sn*-glycerol (10a')

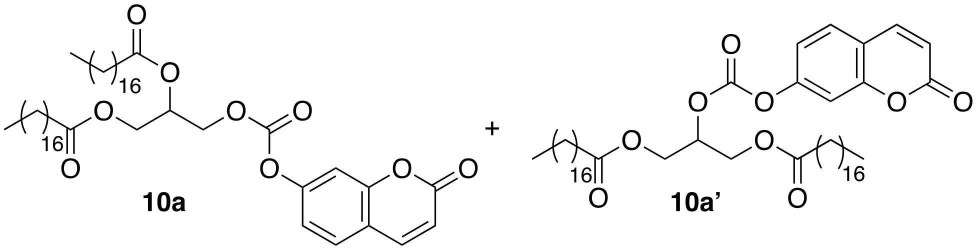

10a:**10a'** 72:28

Molecular formula: $C_{49}H_{80}O_9$
Molecular Mass: 812.5802 g/mol
Physical appearance: white solid
TLC: *Rf* (silica gel) = 0.68 (*n*-pentane/EtOAc 2:1); UV (254nm) and KMnO4 active.
^{1}H NMR (400 MHz, $CDCl_3$), δ (ppm): 7.69 (d, *J* = 9.6 Hz, 1H, CH), 7.50 (d, *J* = 8.4 Hz, 1H, CH), 7.23 (d, *J* = 2.2 Hz, 1H, CH), 7.14 (dt, *J* = 8.6 Hz, *J* = 2.2 Hz, 1H, CH), 6.41 (d, H, *J* = 9.4 Hz, 1H, CH), 5.39-5.34 (m, 1H, CH), 4.49 (dd, *J* = 11.8 Hz, *J* = 3.8 Hz, 1H, CH_2), 4.38-4.33 (m, 2H, CH_2), 4.22 (dd, *J* = 11.5 Hz, *J* = 5.5 Hz, 1H, CH_2), 2.36-2.31 (m, 4H, 2 x CH_2), 1.65-1.58 (m, 4H, 2 x CH_2), 1.32-1.23 (m, 56H, 28 x CH_2), 0.89-0.85 (m, 6H, 2 x CH_3).
^{13}C NMR: (100 MHz, $CDCl_3$), δ (ppm): 173.8, 173.1, 172.8, 160.0, 154.6, 153.1, 152.5, 142.5, 128.6, 117.5, 116.8, 116.3, 109.8, 68.3, 66.8, 64.9, 61.6, 34.1, 34.0,

33.9, 31.8, 29.6, 29.6, 29.5, 29.5, 29.4, 29.4, 29.3, 29.2, 29.2, 29.0, 29.0, 24.8, 22.6, 14.0.

HRMS (ESI^+) m/z: calcd. for $[M+Na]^+$ = $[C_{49}H_{80}O_9Na]^+$: 835.56946; found: 835.56945.

5. NMR spectra

^{1}H NMR of 2

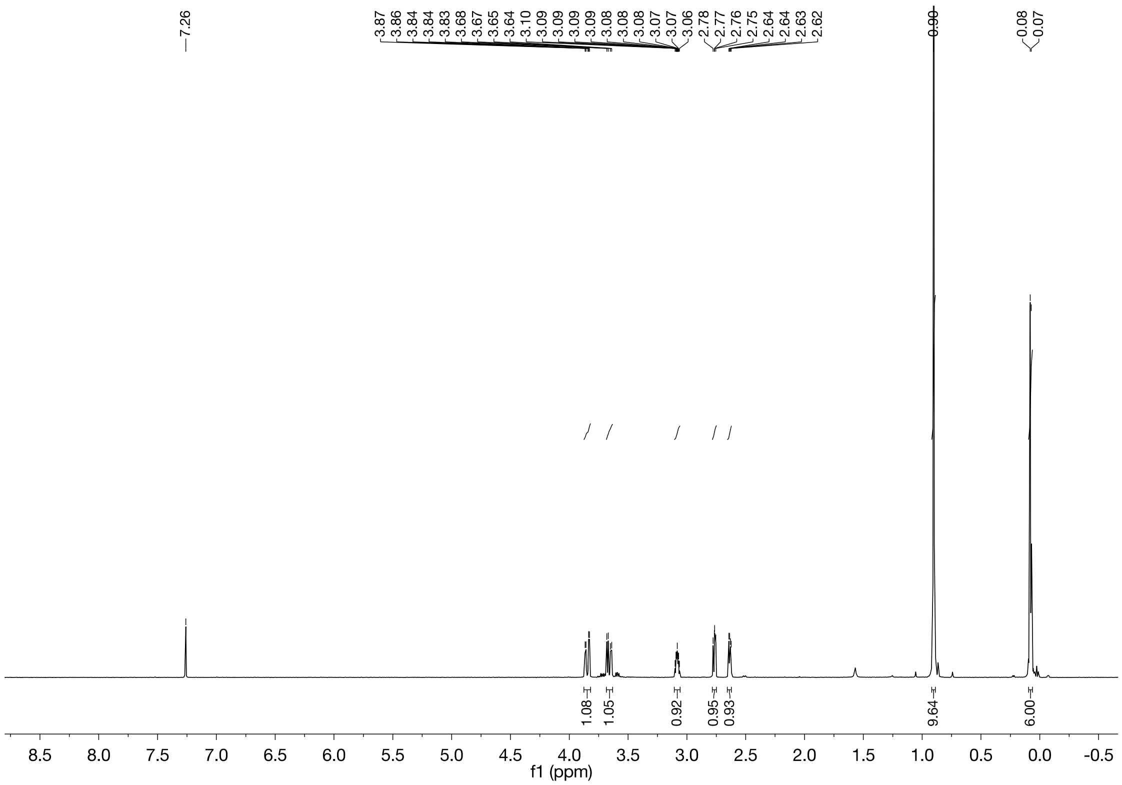

^{13}C NMR of 2

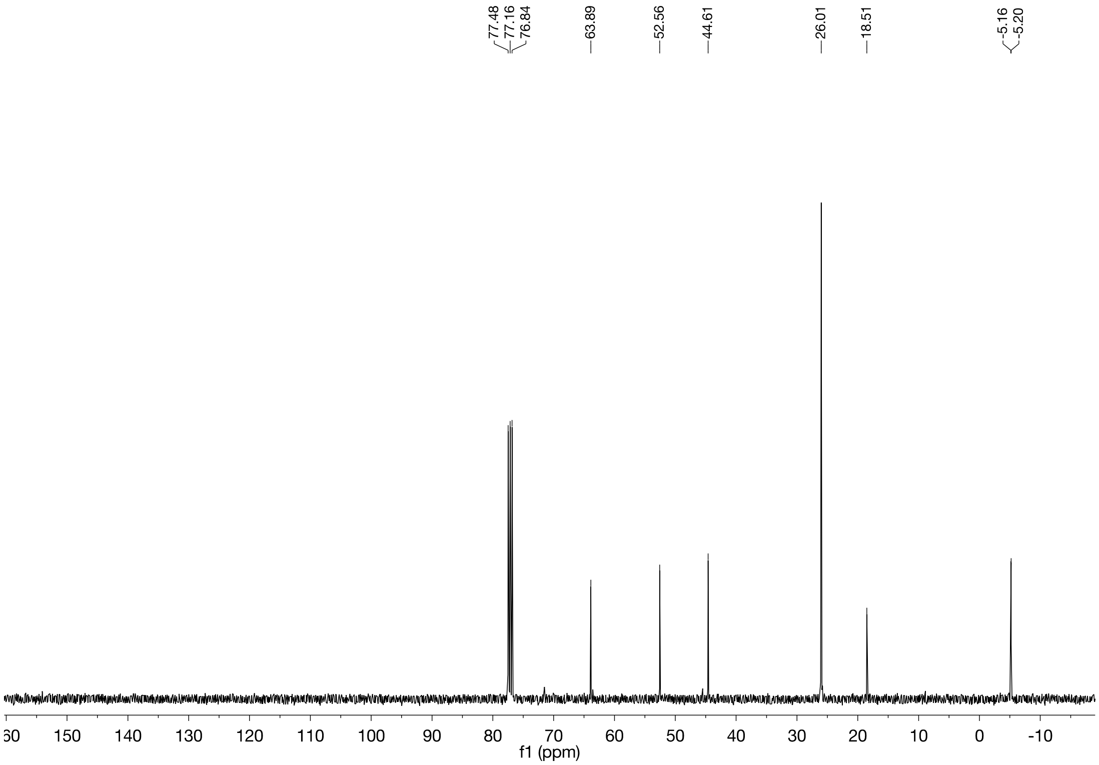

^{1}H NMR of 4a

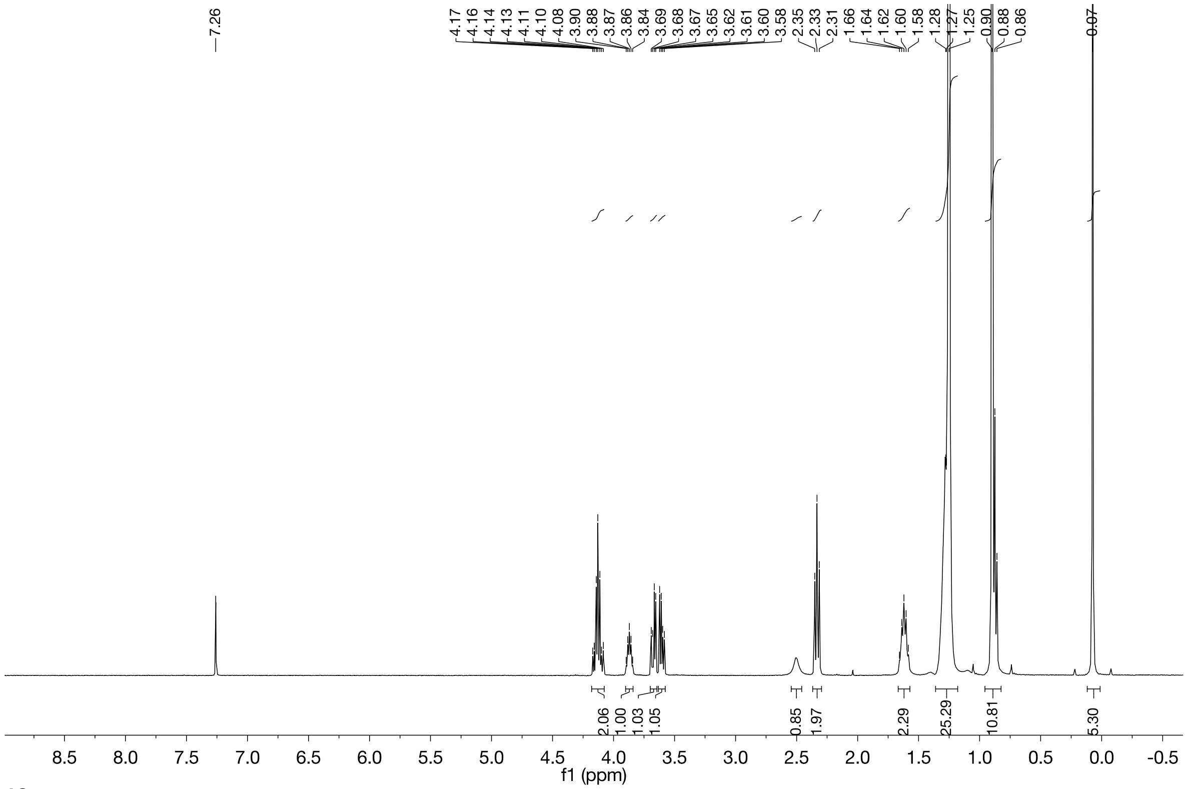

^{13}C NMR of 4a

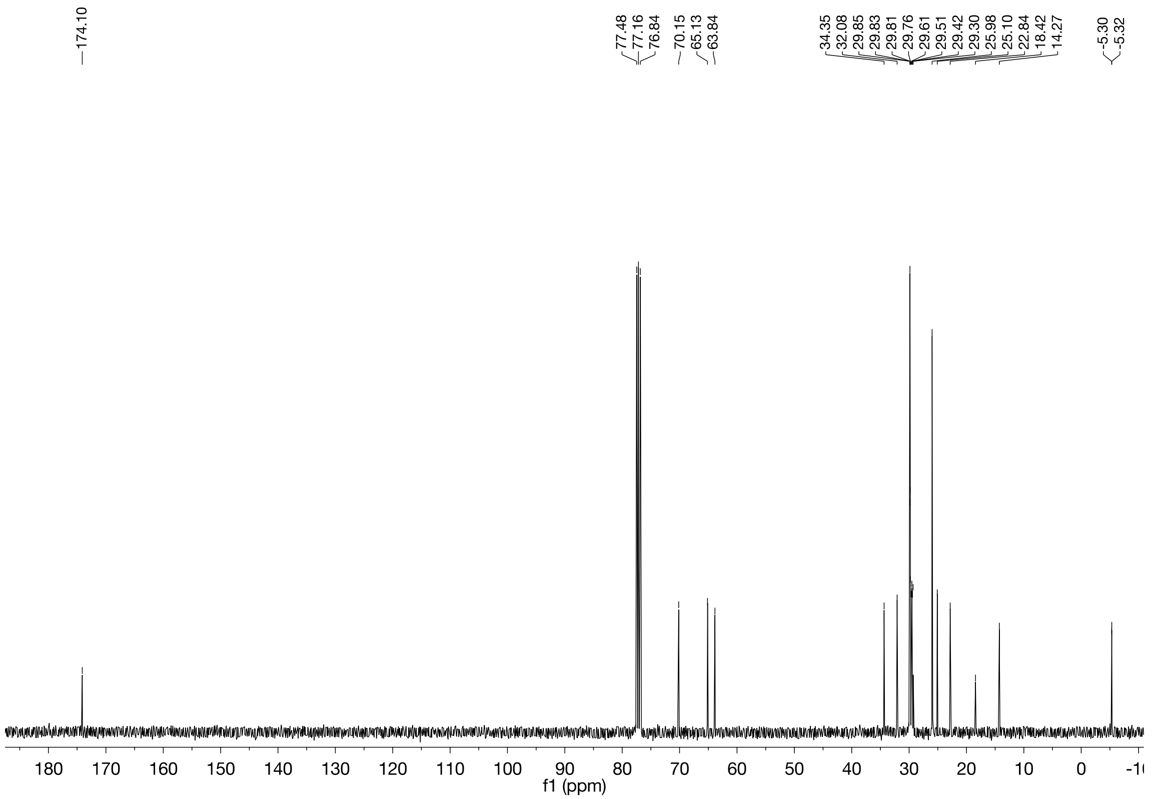

^{1}H NMR of 5a

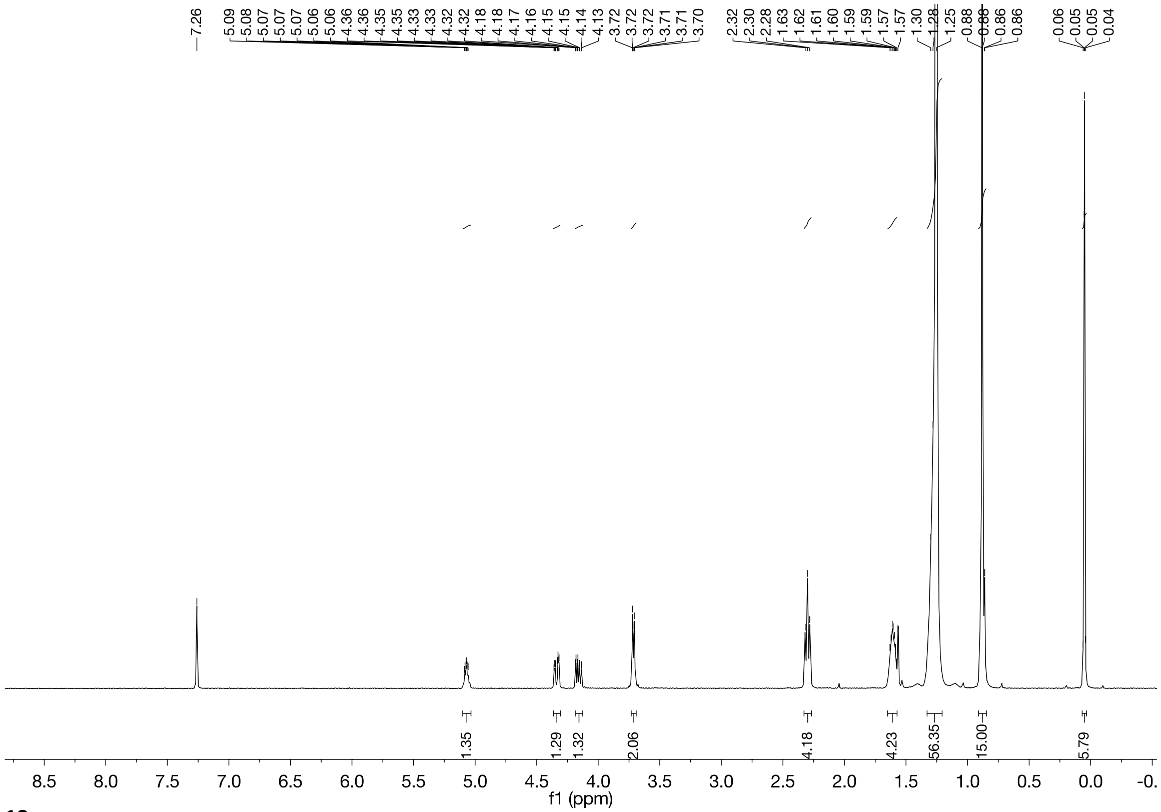

^{13}C NMR of 5a

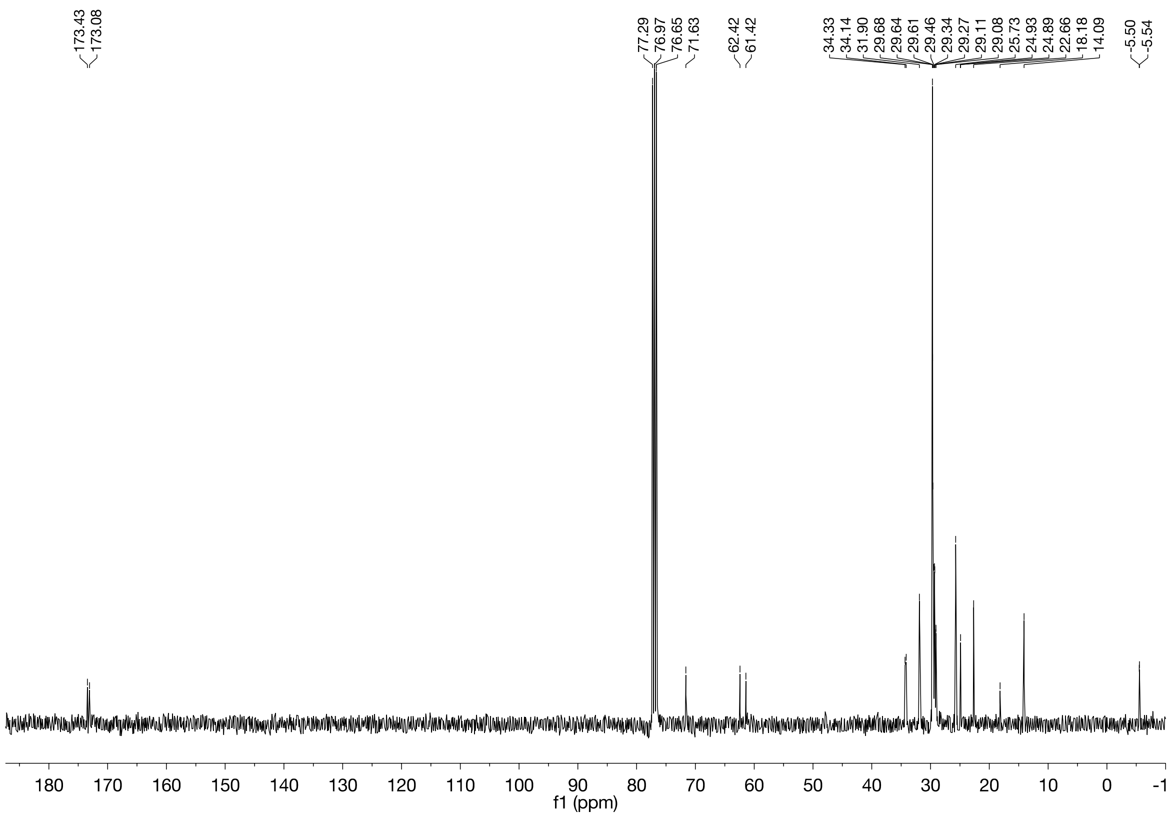

^{1}H NMR of 5b

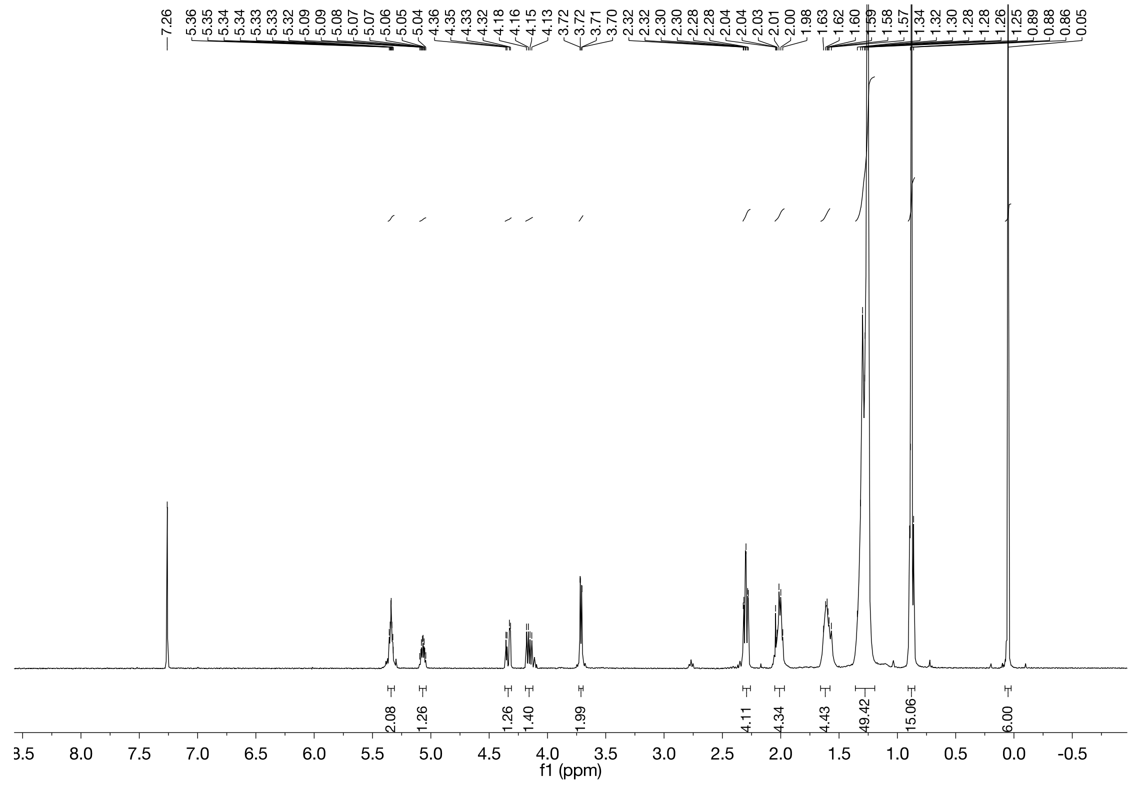

^{13}C NMR of 5b

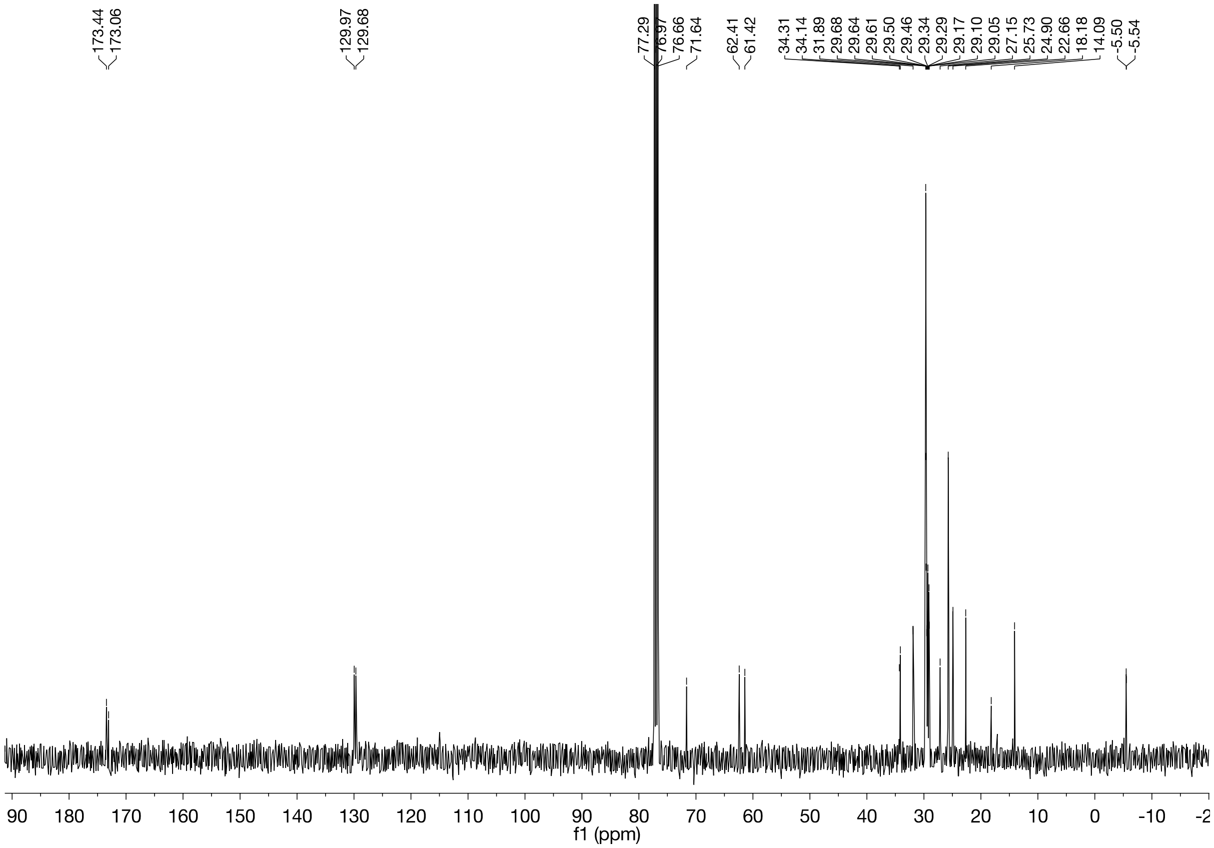

^{1}H NMR of 5c

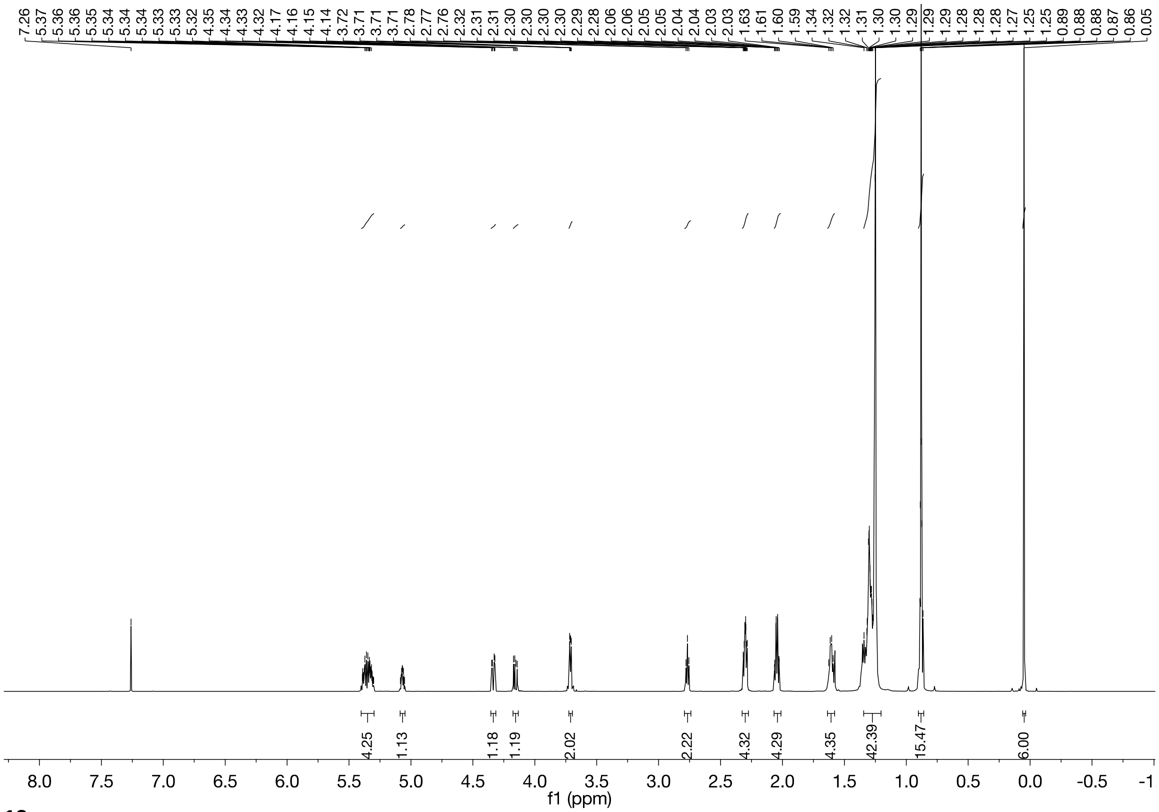

^{13}C NMR of 5c

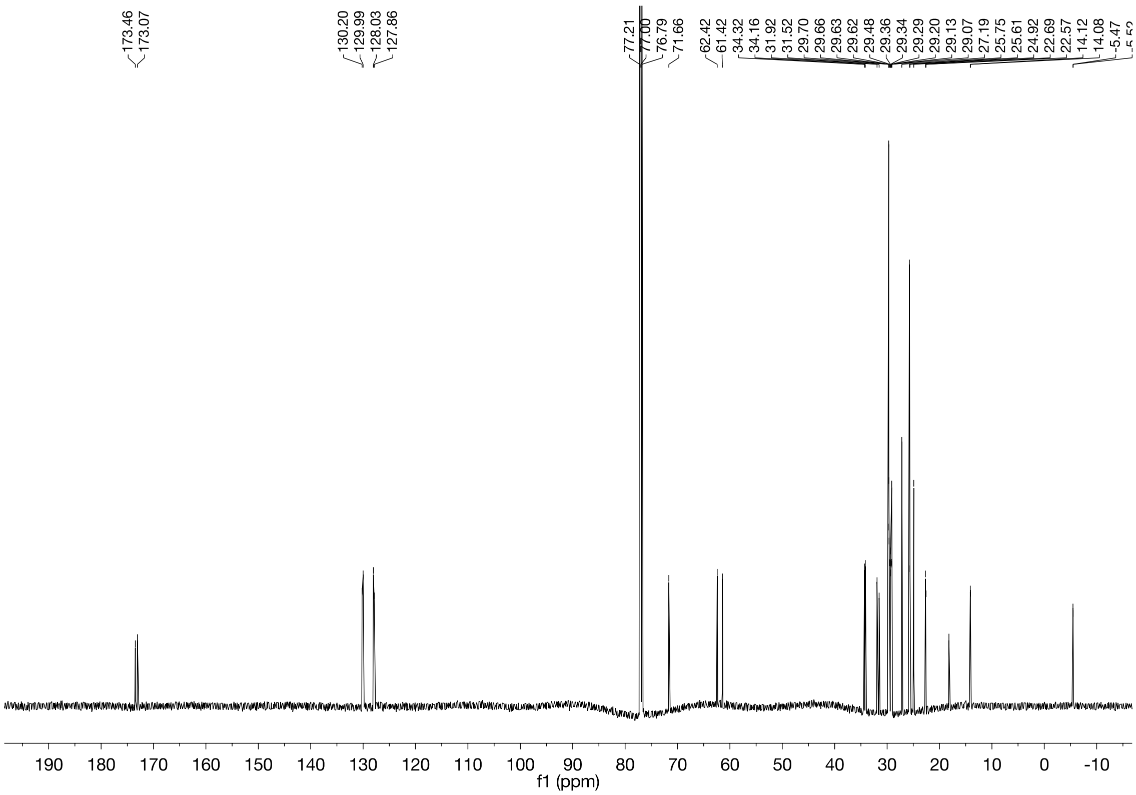

^{1}H NMR of 5d

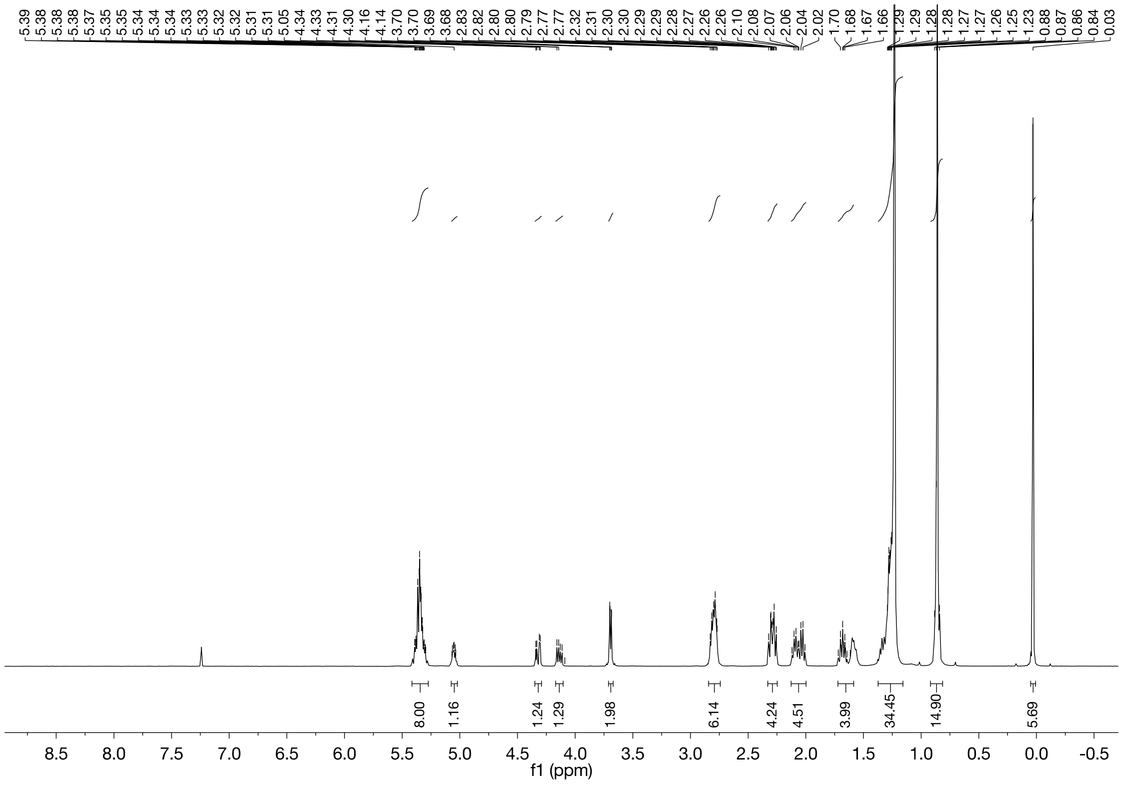

^{13}C NMR of 5d

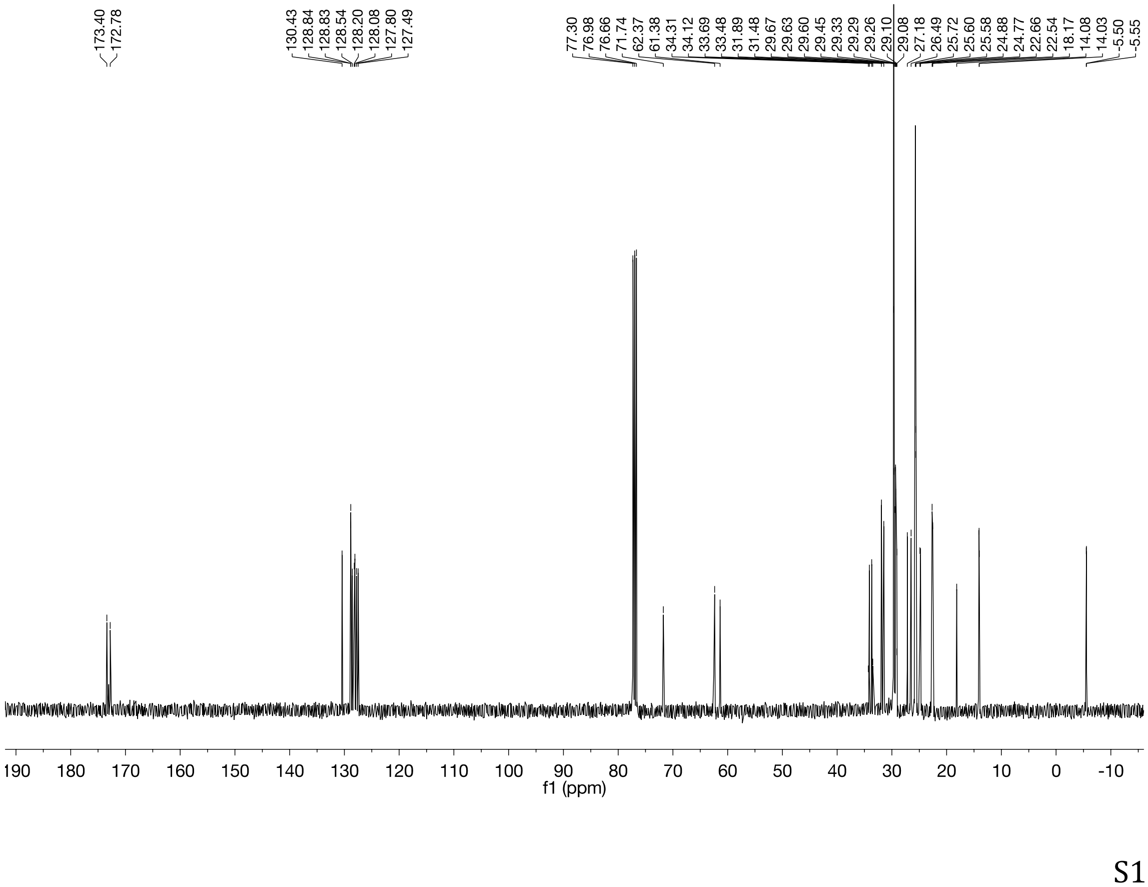

^{1}H NMR of 6a

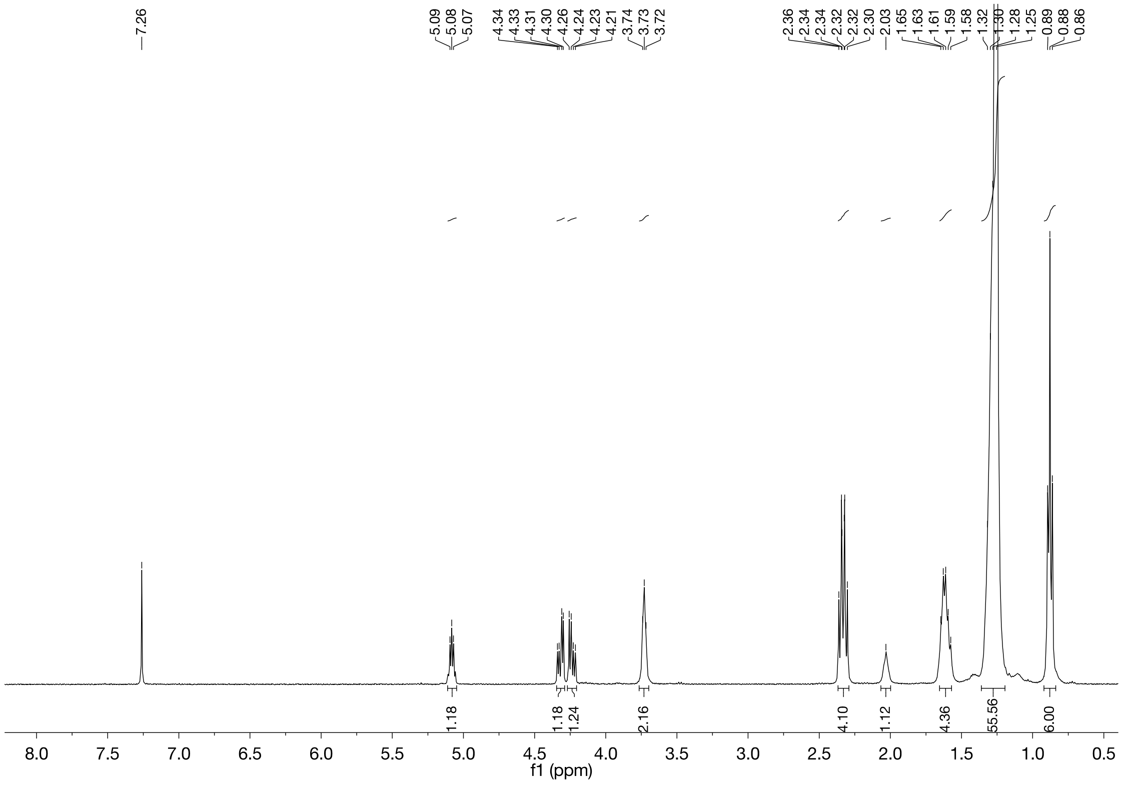

^{13}C NMR of 6a

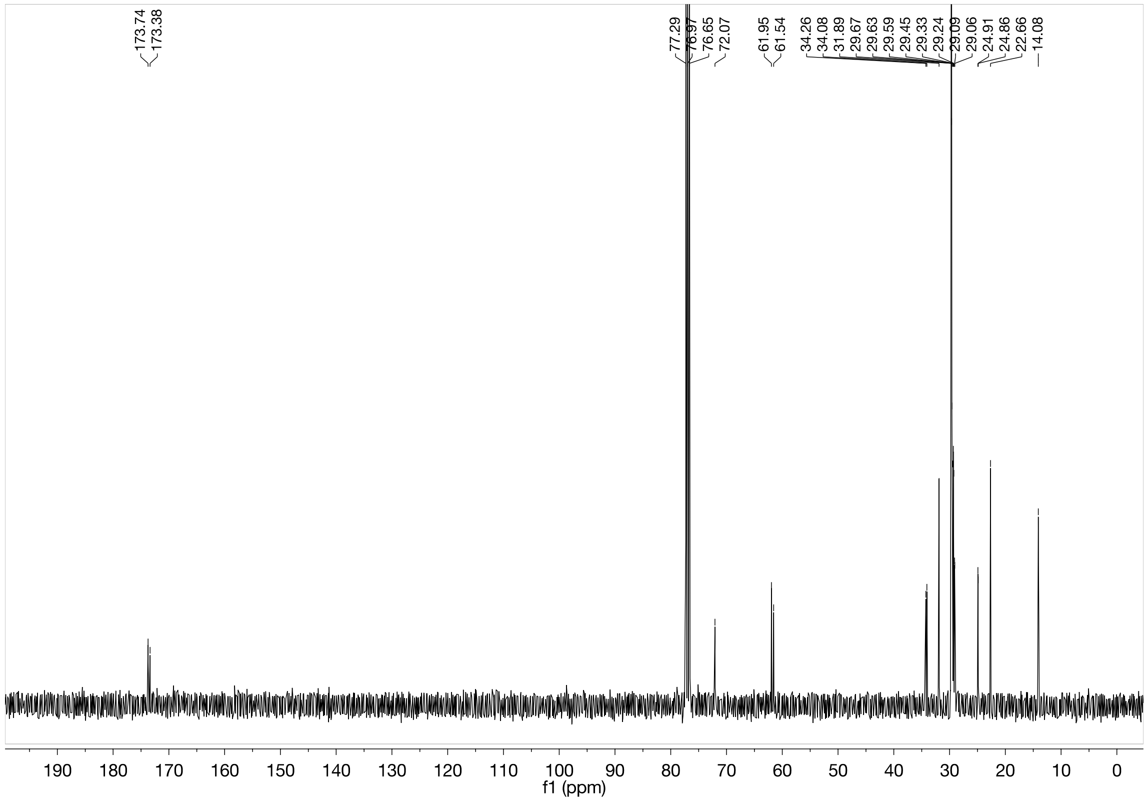

^{1}H NMR of 8a

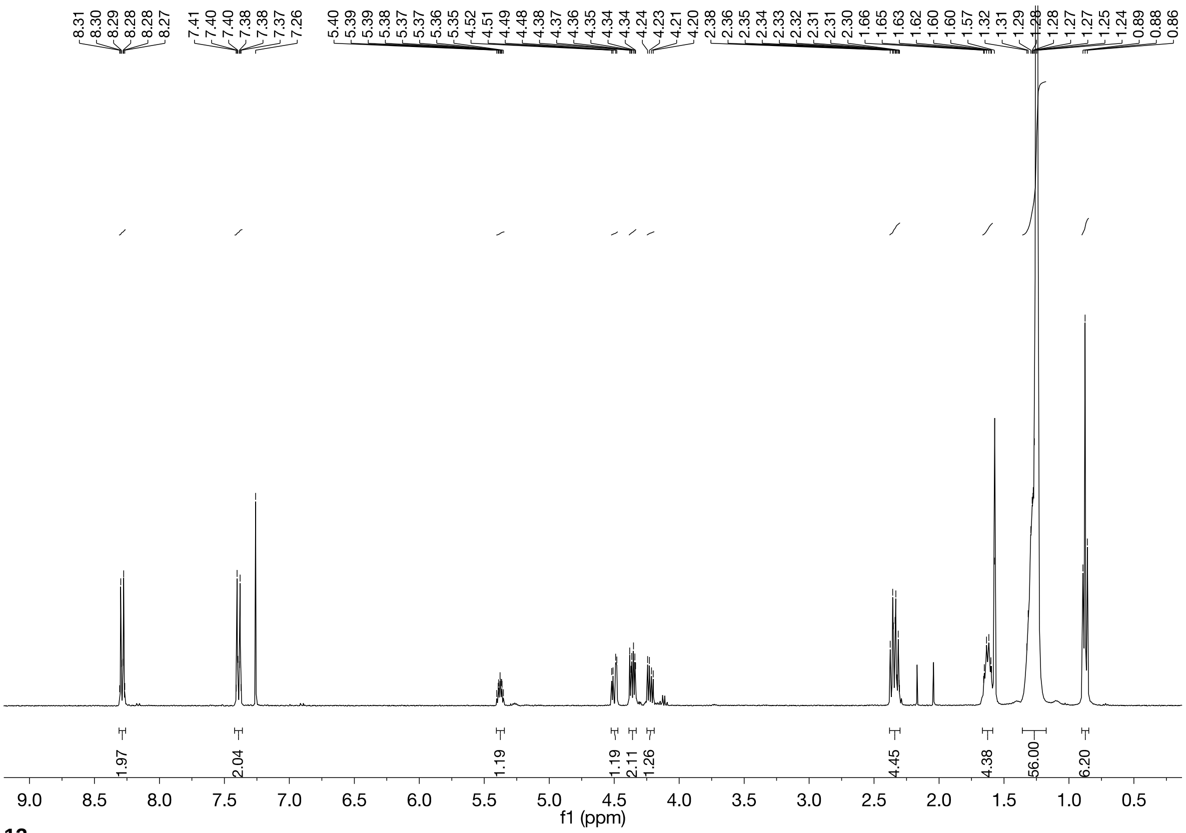

^{13}C NMR of 8a

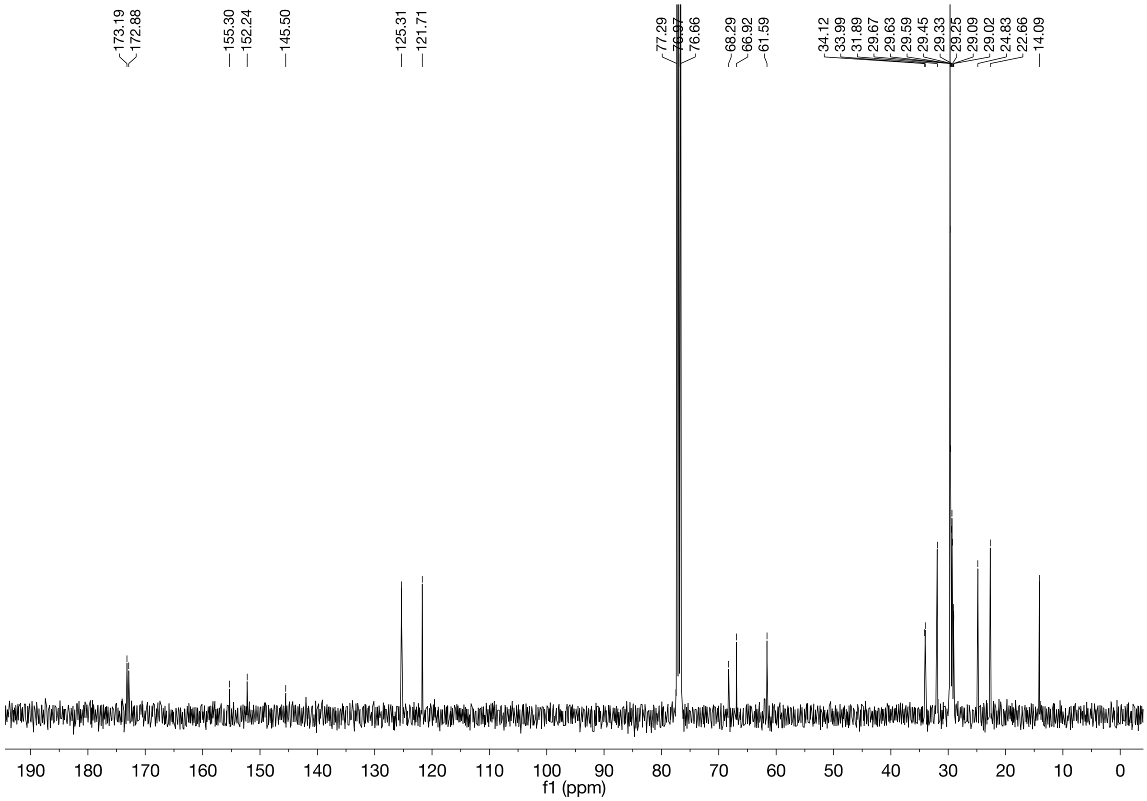

^{1}H NMR of 10a and 10a’

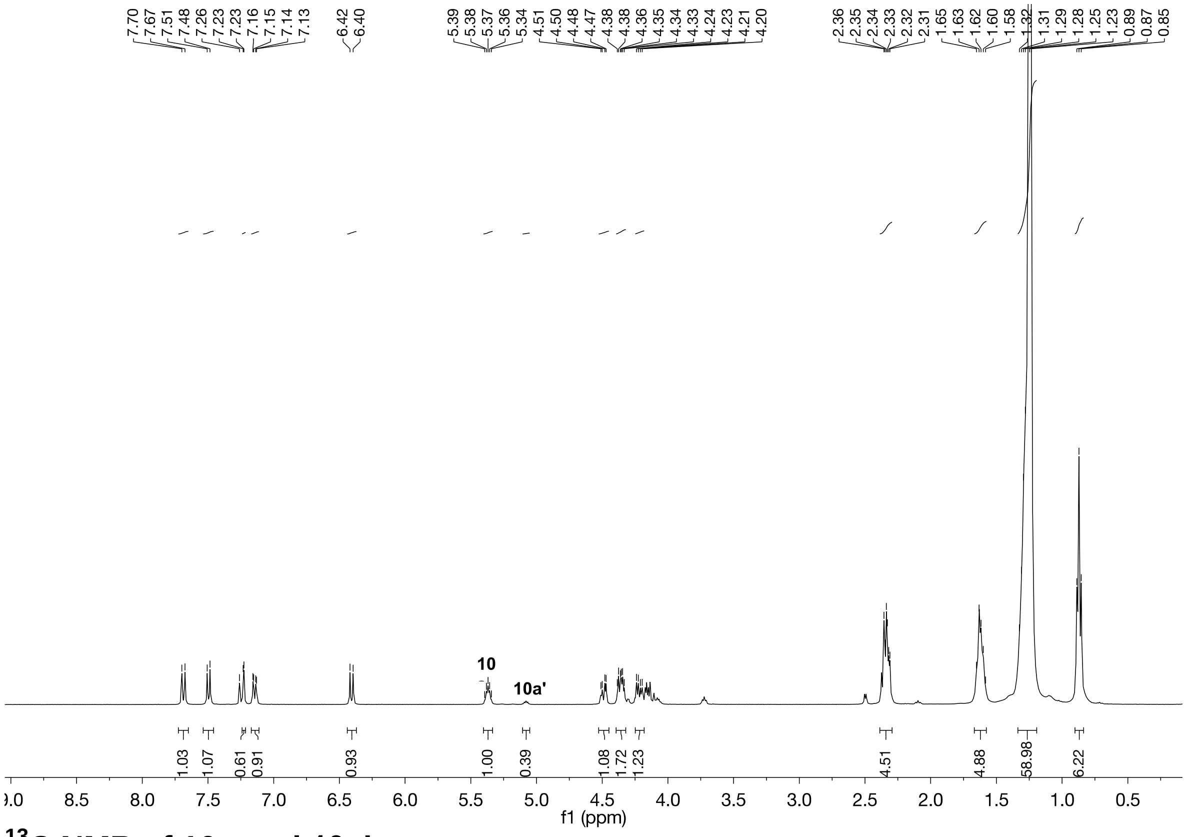

^{13}C NMR of 10a and 10a’

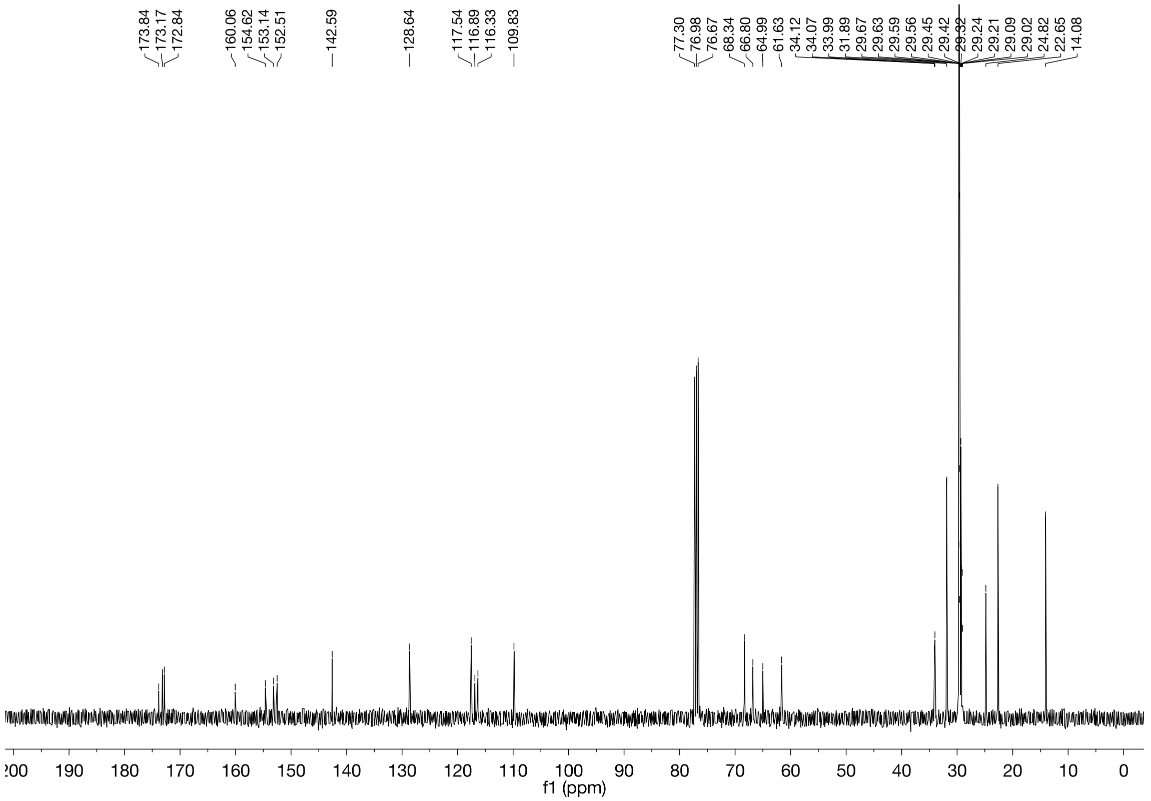

6. Chiral stationary phase high-performance liquid chromatography (CSP-HPLC) analysis.

In order to determine the enantiomeric excess of the MAG **4a**, the reaction was repeated using a close related glycidol derivative **11** under otherwise identical conditions (Section 2.2). Analysis of the corresponding MAG **12** by High-Performance Liquid Chromatography-Chiral Stationary Phase (CSP-HPLC) analysis showed an enantiomeric excess of 17%.

Analysis of *rac*-**12**. CSP-HPLC conditions OD-H; *n*-heptane/iPrOH 98:2, 0.7mL/min

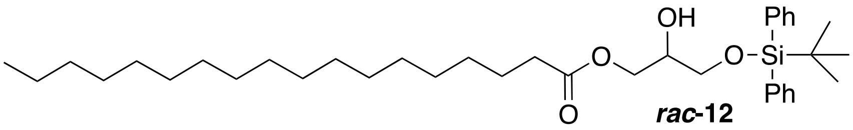

***rac*-12**

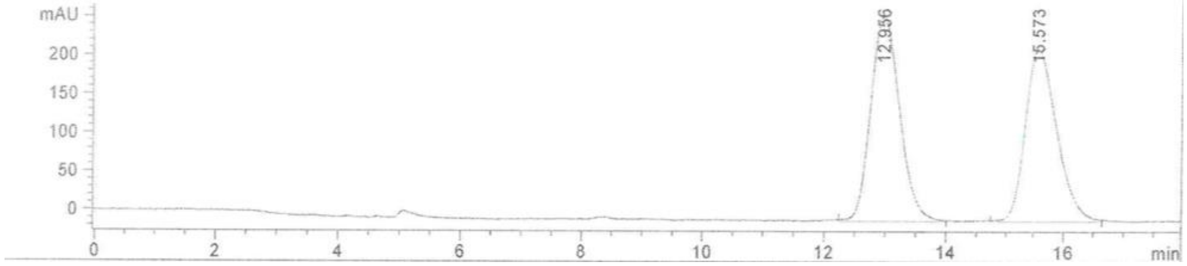


```
Signal 2: DAD1 C, Sig=210,8 Ref=360,100

Peak RetTime Type  Width     Area        Height      Area
  #   [min]        [min]   [mAU*s]       [mAU]        %
----|-------|----|-------|----------|----------|--------|
   1  12.956 VV    0.4851 8785.63574  264.75421  50.7734
   2  15.573 VV    0.5065 8517.99121  222.58554  49.2266

Totals :                  1.73036e4   487.33975
```

Analysis of MAG **12** obtained using the protocol described in Section 2.2.

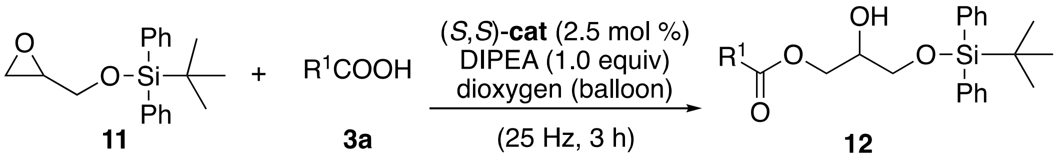

11 **3a** **12** **ee 17%**

OD-H; *n*-heptane/iPrOH 98:2, 0.7 mL/min

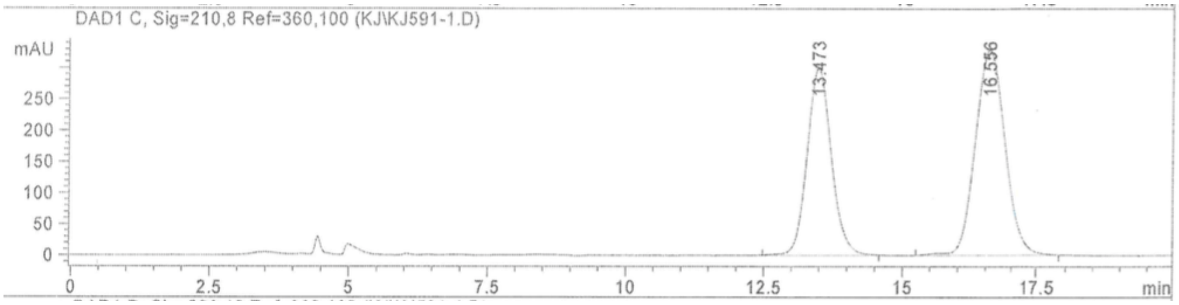

Signal 2: DAD1 C, Sig=210,8 Ref=360,100

Peak #	RetTime [min]	Type	Width [min]	Area [mAU*s]	Height [mAU]	Area %
1	13.473	BB	0.4711	9162.28516	299.37354	41.6188
2	16.556	BB	0.6058	1.28525e4	328.95532	58.3812
Totals :				2.20148e4	628.32886	

7. References

1. Fodran, P.; Minnaard, A. J. *Org, Biomol. Chem.* **2013**, *11*, 6919−6928.
2. Wang, L.; Thai, K.; Gravel, M. *Org. Lett.* **2009,** *11,* 891−893.
3. Burgos, C. E.; Ayer, D. E.; Johnson, R. A. *J. Org. Chem.* **1987,** *52*, 4973−4977.
4. Burgula, S.; Swarts, B.M.; Guo, Z. *Chem. Eur. J.* **2012**, *18,* 1194−1201.
